# Study on Walnut Oil–Fructooligosaccharide Emulsions Fabricated by High-Pressure Microfluidization and Their Application in Improving Rice Quality

**DOI:** 10.3390/foods15162885

**Published:** 2026-08-18

**Authors:** Maman Baligen, Zhiqiang Lu, Ruoxi Wei, Yansong Gao, Qiang Ma, Zhenchao La, Tulehanjiang Dilare, Tuoheti Ayiguli, Haowen Liu, Lingming Kong

**Affiliations:** College of Food Science and Pharmaceutics, Xinjiang Agricultural University, Urumqi 830002, China; 18199145179@163.com (M.B.); lzq0215@xjau.edu.com (Z.L.); 13363967202@163.com (R.W.); 19703538864@163.com (Y.G.);

**Keywords:** high-pressure microfluidization, walnut oil, fructooligosaccharide, emulsion stability, cooked rice quality, emulsions

## Abstract

The present study aimed to optimize the fabrication conditions of walnut oil–fructooligosaccharide emulsion and evaluate its efficacy in improving the quality of cooked rice. Three food-grade emulsifiers, namely sucrose fatty acid ester (SE), polyglycerol fatty acid esters (PGEs), and diacetyl tartaric acid ester of mono- and diglycerides (DATEM), were adopted to investigate the influences of water–oil ratio, emulsifier dosage, and high-pressure microfluidization (HPM) pressure on emulsion indicators including the emulsion stability index (ESI), particle size, polydispersity index (PDI), zeta potential, micromorphology, and storage stability. The results reveal that SE exhibited the superior emulsifying capacity, with the optimal water–oil ratio and SE dosage determined to be 6:4 and 4% (*w*/*w*, based on oil mass), respectively. HPM treatment further reduced droplet size and elevated the ESI, and 150 MPa was identified as the optimal homogenization pressure. Subsequently, the optimized emulsion was applied during rice cooking, with water, pure walnut oil, pure fructooligosaccharide (FOS), simple physical mixture of the two raw materials and crude emulsion set as parallel control groups. Compared with all control treatments, rice cooked with 150 MPa homogenized emulsion possessed the minimum hardness value of 3297.22 and the maximum springiness of 0.74, accompanied by the optimal water retention capacity, luminosity, and the most abundant volatile organic compounds (VOCs). In conclusion, walnut oil–fructooligosaccharide emulsion fabricated via HPM exerted synergistic improvements on the texture and flavor of cooked rice, which could provide a technical reference for the functional modification of staple foods.

## 1. Introduction

Cooked rice, the dominant staple food in the daily diet of Chinese residents, has long been a research focus in the food industry regarding its eating quality and processing properties. Conventional cooked rice suffers from prominent drawbacks including rapid starch retrogradation, hardening during storage, and monotonous flavor, which severely restrict its palatability and shelf-life extension. With the continuous improvement of public health awareness, the development of functional cooked rice with dual functions of nutritional fortification and quality improvement has become a vital direction for the transformation and upgrading of the staple food industry.

Walnut oil is abundant in bioactive constituents such as polyunsaturated fatty acids (primarily linoleic acid and α-linolenic acid), phenolic compounds (gallic acid, ellagic acid, etc.), phytosterols, squalene, and melatonin. These bioactive components jointly endow walnut oil with multiple health-promoting bioactivities, including anti-inflammatory, anti-tumor, and immunomodulatory effects [1,2]. FOS acts as a crucial substrate in the human digestive tract, facilitating the proliferation of beneficial intestinal microorganisms by stimulating the synthesis of short-chain fatty acids. Prebiotics exhibit prominent benefits in regulating intestinal microflora, boosting mineral absorption, and alleviating diabetes, allergies and metabolic disorders, which has aroused growing research interest in their pharmaceutical and food applications; multiple prebiotic supplements have been made commercially available on the market [3]. The walnut oil–fructooligosaccharide delivery system constructed by combining the two ingredients is expected to uniformly disperse lipophilic bioactive components in the aqueous phase while simultaneously delaying starch retrogradation, optimizing texture properties, and enhancing the nutritional value of cooked rice.

The stability of food emulsions is the basis for achieving other properties. During their production and processing, emulsions tend to become unstable due to their thermodynamic instability, and it is usually necessary to add emulsifiers to stabilize emulsions [4]. Emulsions fabricated via traditional high-speed shear homogenization generally present disadvantages such as large droplet size, uneven particle distribution and poor long-term storage stability, which cannot satisfy the complex environmental conditions during rice cooking.

High-pressure microfluidization (HPM) represents a distinctive high-pressure homogenization technology that integrates multiple mechanical forces, including high-velocity impact, high-frequency oscillation, instantaneous pressure drop, intense shear rate and hydrodynamic cavitation [5]. As a scalable physical modification technique free of chemical additives, HPM reduces particle size and improves interfacial activity through intensive shear and cavitation effects, thereby enhancing overall emulsion stability [6]. This technology has demonstrated remarkable technical superiority in the fabrication of functional emulsion delivery systems in recent years. Nevertheless, systematic investigations concerning the regulatory effects of emulsifier types and dosages on the physicochemical properties of walnut oil–fructooligosaccharide emulsions, as well as the underlying mechanisms of HPM processing parameters on multi-dimensional emulsion performance, remain insufficient. Furthermore, the practical efficacy and action pathways of such functional emulsions for improving cooked rice quality have not been fully elucidated.

## 2. Materials and Methods

### 2.1. Materials and Reagents

Rice was purchased from Yihai Kerry Food Marketing Co., Ltd. (Baicheng, China). Walnut oil was supplied by Aksu Zhejiang Fruit Industry Co., Ltd. (Aksu, China). Fructooligosaccharide (FOS) was obtained from Shandong Bailong Chuangyuan Biotechnology Co., Ltd. (Dezhou, China). Sucrose fatty acid ester (SE), polyglycerol fatty acid esters (PGEs), and diacetyl tartaric acid ester of mono- and diglycerides (DATEM) were provided by Guangxi Yuanchang Food Technology Co., Ltd. (Liuzhou, China); Zhengzhou Dahe Food Technology Co., Ltd. (Xingyang, China); and Henan Onist Food Co., Ltd. (Xinxiang, China) respectively. Potassium iodide (analytical grade) and iodine crystals (analytical grade) were sourced from Tianjin Zhiyuan Chemical Reagent Co., Ltd. (Tianjin, China) and Tianjin Kaitong Chemical Reagent Co., Ltd. (Tianjin, China), respectively. Hydrochloric acid (analytical grade) was purchased from Urumqi Baihengda Instrument & Equipment Co., Ltd. (Urumqi, China) Nile Blue S19278 and Nile Red S19279 were supplied by Yuanye Biotechnology Co., Ltd. (Shanghai, China). DPPH assay kit (catalog number: M0112A) and ABTS assay kit (catalog number: M0113A) were purchased from Mengxi Biomedical Technology Co., Ltd. (Suzhou, China). Sodium dodecyl sulfate (SDS, analytical grade) was obtained from Xinbot Chemical Co., Ltd. (Tianjin, China).

### 2.2. Instruments and Equipment

A double-inner-pot electric rice cooker (Midea) was manufactured by Midea Living Electrical Appliances Manufacturing Co., Foshan, China. A texture analyzer model, TA-XT.Plus (TPA), was supplied by Stable Micro Systems, Godalming, UK. A color spectrophotometer was purchased from 3NH Technology Co., Ltd., Guangzhou, China. A UV–visible spectrophotometer (model T700) was provided by Persee General Instrument Co., Ltd., Beijing, China. A moisture analyzer (model DSH-16) was sourced from Jinghai Instrument Co., Ltd., Shanghai, China. A dynamic light scattering instrument Zetasizer Nano ZS was produced by Malvern Panalytical Ltd., Malvern, UK. A laser scanning confocal microscope (CLSM) (LSM 900) was supplied by Carl Zeiss AG, Oberkochen, Germany. A FlavourSpec^®^ flavor analyzer was adopted for volatile compound detection. A biological microscope model, S550T-720HD, was purchased from Suzhou Beitejia Optoelectronics Technology Co., Ltd. (Suzhou, China). A magnetic stirrer (model 78-1) was obtained from Jinyi Instrument Technology Co., Ltd. (Changzhou, Jiangsu, China). A high-speed homogenizer model, FSH-2A, was supplied by Anqing Jiejia Instrument Equipment Co., Ltd. (Shanghai, China). A JN Mini Pro high-pressure microfluidizer was provided by Juneng Nano Biotechnology Co., Ltd., Guangzhou, China. A multifunctional microplate reader (Model KD-810C) was provided by Persee General Instrument Co., Ltd., Beijing, China.

### 2.3. Preparation of Emulsions with Different Water–Oil Ratios

Three food-grade emulsifiers, namely SE, PGEs, and DATEM, were selected for this experiment. Briefly, 1.5 g of FOS was accurately weighed and dissolved in an appropriate amount of deionized water to prepare an aqueous solution with a mass fraction of 1%. The solution was continuously stirred with a magnetic stirrer at room temperature for 2 h to achieve full dissolution and uniform dispersion, followed by standing overnight (24 h) to serve as the aqueous phase.

Each of the three emulsifiers was separately incorporated into the aqueous phase at a dosage of 3% based on the mass of the oil phase, and magnetic stirring was performed until complete dissolution. Subsequently, walnut oil was slowly added into the prepared aqueous phase at water–oil volume ratios of 9:1, 8:2, 7:3, 6:4, and 5:5, respectively. The mixtures were homogenized using a high-speed homogenizer at 13,000 r/min for 5 min to obtain crude emulsions.

The emulsifying capacities of the three emulsifiers under various water–oil ratios were systematically compared. A comprehensive evaluation method was further adopted to screen out the emulsifier and water–oil ratio with the optimal overall emulsifying performance.

### 2.4. Preparation of Emulsions with Different Emulsifier Dosages

On the basis of Section 2.3, the influences of different dosages of the emulsifier with the optimal emulsifying performance on emulsion properties were explored. The optimal water–oil ratio confirmed in Section 2.3 was fixed, and the emulsifier was added at 1%, 2%, 3%, 4% and 5% by mass of the oil phase, respectively. The emulsion preparation procedures were consistent with those described in Section 2.3.

### 2.5. Preparation of Emulsions Treated Under Different HPM Pressures

After the optimal emulsifier, the water–oil ratio and emulsifier dosage were determined in Section 2.3 and Section 2.4, and crude emulsions were prepared via high-speed homogenization. Subsequently, the obtained crude emulsions were subjected to HPM treatment at pressures of 30 MPa, 60 MPa, 90 MPa, 120 MPa and 150 MPa, respectively, to acquire homogenized emulsions under various processing pressures.

### 2.6. Determination of ESI

The measurement method was slightly modified based on the protocol reported by Li et al. [7]. Briefly, 50 μL of emulsion treated by high-speed homogenization or high-pressure microfluidization was withdrawn from the bottom of the beaker and mixed with 5 mL of 1% SDS aqueous solution. A pure 1% SDS solution was set as the blank control. The absorbance value of the mixture was measured at 500 nm using a spectrophotometer. After the emulsion was kept standing for 10 min, absorbance was re-detected following the identical operation procedure. The calculation formula is shown in Equation (1):

(1) $$ESI=\frac{10\times A_{0}}{A_{0}-A_{10}}\times 100$$

where *A*_0_ represents the absorbance of the emulsion at 0 min, and *A*_10_ denotes absorbance after a 10 min reaction between the emulsion and SDS solutions.

It should be noted that this 10 min ESI measurement serves as a rapid screening indicator for comparative evaluation of initial emulsifying activity among different treatments. This parameter does not represent the absolute long-term storage stability of the emulsions. The long-term physical stability was independently assessed by the creaming index (CI) during 20 days of storage, as described in Section 2.14.

### 2.7. Confocal Laser Scanning Microscopy (CLSM)

An appropriate amount of emulsion sample was transferred into a centrifuge tube, followed by the addition of Nile Blue dye solution at a concentration of 20 μg/mL. The mixture was incubated in the dark for 30 min. Subsequently, Nile Red dye solution (1 μM) was supplemented, and another 30 min of incubation was carried out under light-shielded conditions. After the staining procedure was completed, a drop of stained sample was placed on a glass slide, and microscopic images were captured at 400× magnification under the CLSM.

### 2.8. Macroscopic and Microscopic Morphology of Emulsions

(1) Macroscopic observation: Freshly prepared emulsions were transferred into transparent serum vials. The overall appearance was photographed, and the macroscopic state of each emulsion was visually evaluated.

(2) Microscopic observation: A volume of 10 μL of fresh emulsion was diluted 100-fold with deionized water. The diluted sample was observed under a 40× objective lens to characterize the microscopic droplet morphology.

### 2.9. Determination of PDI

The droplet size and zeta potential of emulsions were determined by dynamic light scattering (DLS) using a Malvern Zetasizer Nano ZS instrument (Malvern Panalytical Ltd., Malvern, UK). For droplet size measurement, the emulsion sample was diluted four times, and 1 mL of the diluted sample was transferred into a measurement cell. After thermal equilibration at 25 °C for 120 s, the droplet size distribution and PDI value were recorded, with three replicate measurements performed for each sample.

### 2.10. Determination of Zeta Potential

For zeta potential measurement, 1 mL of undiluted emulsion sample was loaded into a zeta potential U-cell. The electrodes of the U-cell were aligned with the sample electrodes, followed by thermal equilibration at 25 °C for 120 s prior to recording zeta potential values, with three replicates tested per sample.

### 2.11. DPPH Radical Scavenging Activity

Exactly 20 μL of the prepared sample solution was mixed thoroughly with 380 μL of DPPH working solution, and the mixture was incubated at room temperature in the dark for 20 min. After incubation, 200 µL of the supernatant was transferred to a 96-well microplate, and absorbance was detected at 515 nm using a microplate reader. The extraction solvent was used as the blank control group. The DPPH radical scavenging rate of the sample was calculated via Equation (2):

(2) $$DPPH\;free\;radical\;scavenging\;rate\left(\%\right)=\frac{A_{0}-A_{1}}{A_{0}}.$$

where *A*_0_ is the absorbance of the blank group, and *A*_1_ represents the absorbance of the sample.

### 2.12. ABTS^+^ Radical Scavenging Activity

An accurate amount of 10 μL of sample solution was mixed with 190 μL of ABTS^+^ working solution and fully blended, followed by standing at room temperature for 20 min without generating sediment or turbidity. Afterwards, 200 μL of the supernatant was transferred into a 96-well plate, and absorbance was determined at 734 nm using a microplate reader. The extraction solution was used as the blank control. The ABTS^+^ radical scavenging capacity of samples was calculated according to Equation (3):

(3) $${ABTS}^{+}\;free\;radical\;scavenging\;rate\left(\%\right)=\frac{A_{0}-A_{1}}{A_{0}}$$

where *A*_0_ is the absorbance of the blank group, and *A*_1_ represents the absorbance of the sample.

### 2.13. Light Transmittance of Emulsions

The measurement method was slightly modified based on the protocol reported by Wang et al. [8]. The emulsions were diluted 100-fold with deionized water, and the light transmittance of diluted emulsions was determined at a wavelength of 670 nm using a UV–visible spectrophotometer.

### 2.14. Creaming Index (CI) (Storage Stability for 20 Days)

Freshly prepared emulsions were transferred into transparent serum vials with a total liquid height of 3.5 cm. The samples were stored at room temperature for 1, 5, 10, 15 and 20 days, respectively. The macroscopic state of emulsions was observed, and the CI was calculated according to Equation (4) [9]. All measurements were performed in triplicate.

(4) $$CI\left(\%\right)=\frac{H_{c}}{H_{t}}$$

where *H_c_* denotes the height of the transparent serum layer at the bottom, and *H_t_* represents the total height of the emulsion.

### 2.15. Preparation of Cooked Rice

A fixed mass of raw rice was accurately weighed and transferred into an electric rice cooker. Appropriate deionized water was added to wash the rice, and the washing water was completely drained afterwards. Then, the required volume of cooking water was supplemented. Separate groups were supplemented with walnut oil, FOS, a mixture of walnut oil and FOS (not as an emulsion), crude emulsion, and emulsion treated at 150 MPa, respectively, and used for rice cooking. Rice cooked with pure deionized water was set as the blank control group. After 30 min of cooking, cooked rice samples were obtained.

### 2.16. Determination of Moisture Content in Cooked Rice

The moisture content of fresh cooked rice was determined using a DSH-16 moisture analyzer (Jinghai Instrument Co., Ltd., Shanghai, China). The analysis was performed at 105 °C under ambient pressure, and the measurement was terminated automatically when the weight change of the sample was less than 0.005 g over 60 s. All measurements were conducted in triplicate.

### 2.17. Calculation of Water Absorption Rate of Cooked Rice

The water absorption rate of cooked rice was calculated via Equation (5):

(5) $$Water\;absorption\;rate=[(m_{2}-m_{1})/m_{1}]\times 100\%$$

where m_1_ refers to the initial mass of raw rice (50 g), and m_2_ is the mass of cooked rice.

### 2.18. Color Measurement of Cooked Rice

Fresh cooked rice was evenly placed in a Petri dish, and the color parameters were determined using a color spectrophotometer. The luminosity (L*), red–green coordinate (a*), and yellow–blue coordinate (b*) were recorded. Each sample was measured in triplicate, and the average value was adopted. The instrument was calibrated with standard black and white ceramic tiles before each test [10]. The temperature of samples was balanced to the ambient temperature of 25 °C prior to detection.

### 2.19. Texture Measurement of Cooked Rice

A TPA equipped with a P36R probe was adopted. A glass Petri dish with an inner diameter of 9 cm was used for height calibration.

Cooked rice prepared according to the standardized cooking procedure was uncovered immediately after cooking. The rice located approximately 2 cm away from the pot walls and bottom was quickly stirred uniformly in one direction within 10 s using the matched rice spoon. Test samples were collected from the well-mixed rice mass. Exactly 87 g of rice (accurate to 0.5 g) was weighed and transferred into the fixed Petri dish. The rice was flattened evenly with a spoon and compacted with the dish lid, and the lid was then pressed with a 1 kg standard weight for around 5 s; this compressing operation was repeated three times with gentle rotation of the lid before removal.

The Petri dish was placed on the test platform, ensuring the probe was positioned at the central area of the rice sample prior to testing. Each treatment was measured in triplicate under identical conditions, and the average value was recorded.

### 2.20. GC IMS Analysis of Cooked Rice

Exactly 3 g of cooked rice sample was weighed and placed into a 20 mL headspace vial. A total of 20 μL of 100 ppm 2-octanol solution was added as the internal standard. The vial was incubated at 80 °C for 20 min before injection for analysis.

### 2.21. Data Analysis

All experiments were performed in triplicate, and the results are expressed as the mean ± standard deviation. Analysis of variance was performed using IBM SPSS Statistics (version 22.0, IBM Corp., Armonk, NY, USA), and differences were considered significant at *p* < 0.05. Figures were generated using GraphPad Prism (version 10.1.2, GraphPad Software LLC, Boston, MA, USA).

## 3. Results

### 3.1. Screening of Optimal Emulsifier and Water–Oil Ratio

#### 3.1.1. Comparison of ESI of Emulsions Stabilized by Three Types of Emulsifiers

**Figure 1:**
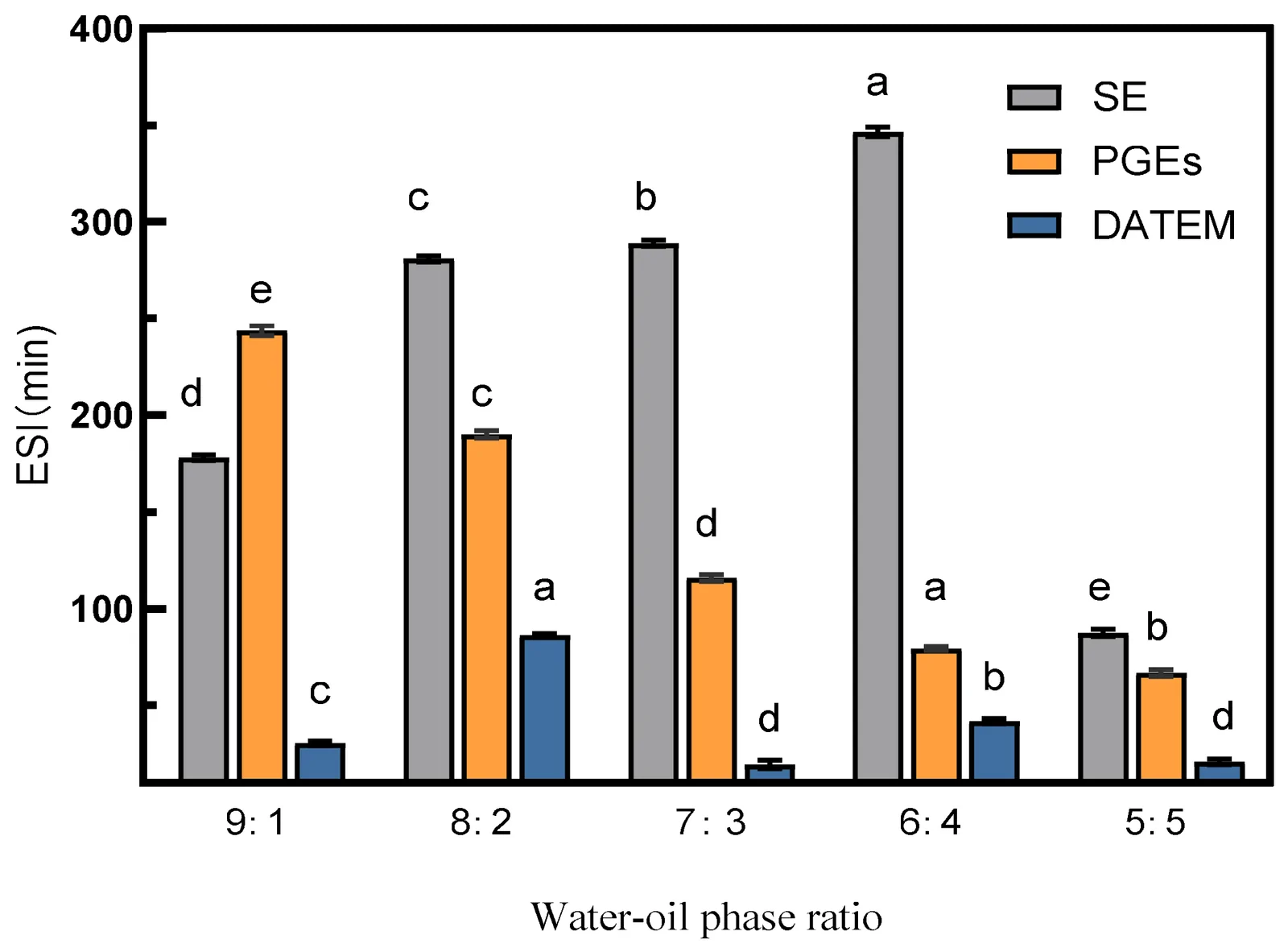
Comparison of emulsion stability index (ESI) of emulsions stabilized by three different emulsifiers. Different lowercase letters above the bars denote significant differences between groups (*p* < 0.05, one-way ANOVA); bars sharing the same letter are not statistically different.

The emulsifying activity was evaluated via the ESI [11]. In this study, the variations in ESI values of oil-in-water (O/W) emulsions stabilized by three emulsifiers (SE, PGEs, and DATEM) at diverse water–oil ratios were investigated, and the corresponding results are presented in Figure 1.

The ESI values of SE-stabilized emulsions first increased and then decreased with the rising proportion of the oil phase, reaching the maximum value at a water–oil ratio of 6:4. This phenomenon could be attributed to the fact that SE molecules were sufficiently adsorbed at the oil–water interface as the oil fraction increased, forming a protective interfacial film to improve the emulsifying activity of the emulsion. This observation is consistent with the report of Tan and Misran [12]. This study pointed out that SE can effectively improve the emulsifying activity of emulsions containing a high oil phase ratio. Nevertheless, a reduction in the ESI was observed when the water–oil ratio was adjusted to 5:5. This may be explained by the emulsifier dosage being insufficient to fully cover the oil droplets, which weakened the interfacial protective film and impaired the overall emulsifying activity. Shao et al. [13] also demonstrated that insufficient emulsifier coverage at the oil–water interface disrupts the integrity of the interfacial film, leading to decreased emulsion activity.

Unlike SE, the ESI values of PGE-stabilized emulsions declined gradually as the oil proportion increased, and the optimal stability was obtained at a water–oil ratio of 9:1, indicating that PGEs perform better in high-water-content systems. Notably, emulsions stabilized by DATEM exhibited significantly lower ESI values across all tested water–oil ratios compared with SE and PGEs, demonstrating the poorest emulsifying activity among the three emulsifiers.

Comprehensive horizontal comparison of the three emulsifiers at identical water–oil ratios revealed distinct activity rankings. At the water–oil ratio of 9:1, the emulsifying activity followed the order of PGEs > SE > DATEM. For water–oil ratios of 8:2, 7:3, 6:4, and 5:5, the activity sequence consistently followed SE > PGEs > DATEM.

#### 3.1.2. Comparison of Macroscopic and Microscopic Morphology of Emulsions Prepared with Three Emulsifiers

**Figure 2:**
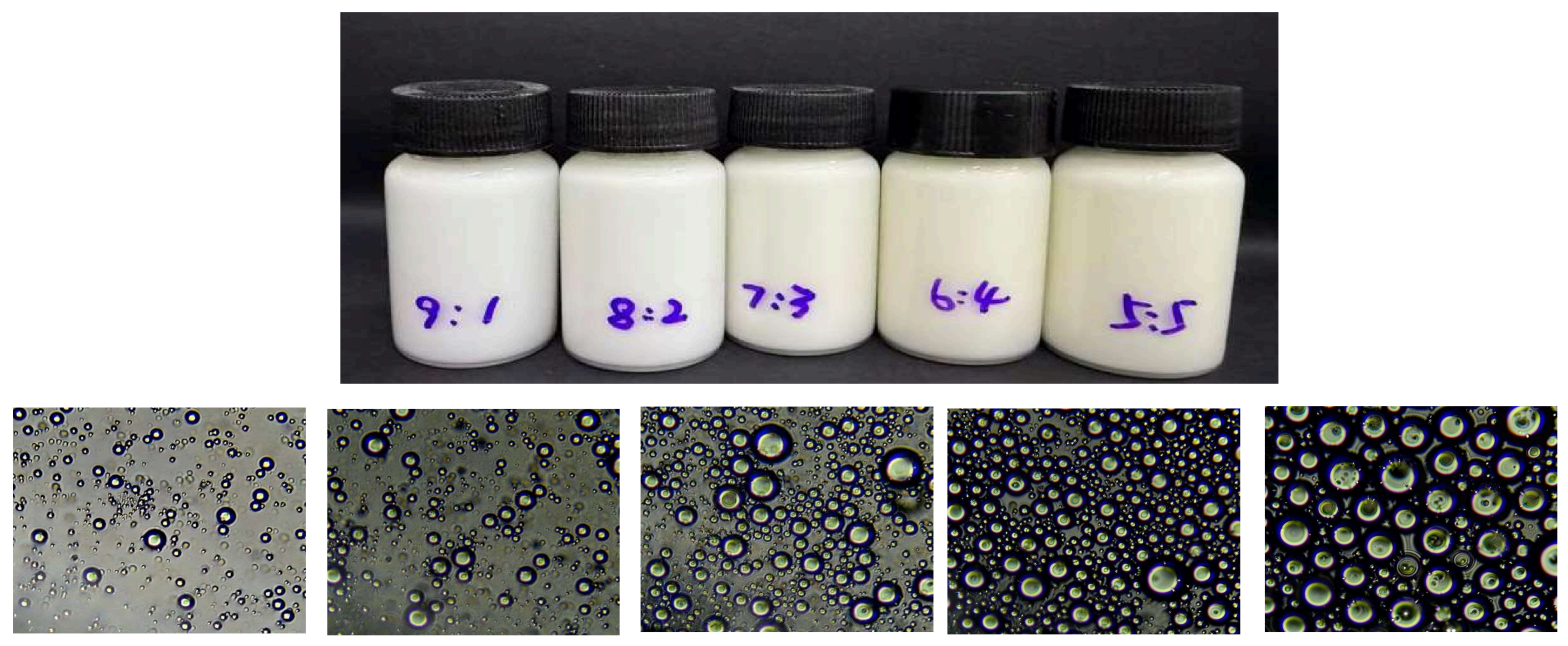
Macroscopic appearance and microscopic morphology of emulsions stabilized by SE. The handwritten content in the figure indicates different water-to-oil phase ratios.

Figure 2 shows the macroscopic appearance and microscopic morphology of oil-in-water emulsions stabilized by SE at different water–oil ratios. Macroscopically, all five groups of emulsions displayed a uniform milky-white appearance without obvious phase separation or water creaming. Corresponding microscopic observations revealed that the droplet size of the dispersed phase gradually increased with the rising oil fraction in the system. The droplets in the microscopic view transformed from sparsely distributed small droplets to densely packed large droplets. This phenomenon indicated that at a fixed SE dosage, the elevated oil fraction increased the total oil volume to be covered by emulsifier per unit volume, facilitating droplet coalescence and growth, which further altered the microscopic droplet size distribution of emulsions. This trend is consistent with the findings reported by Meng [14].

**Figure 3:**
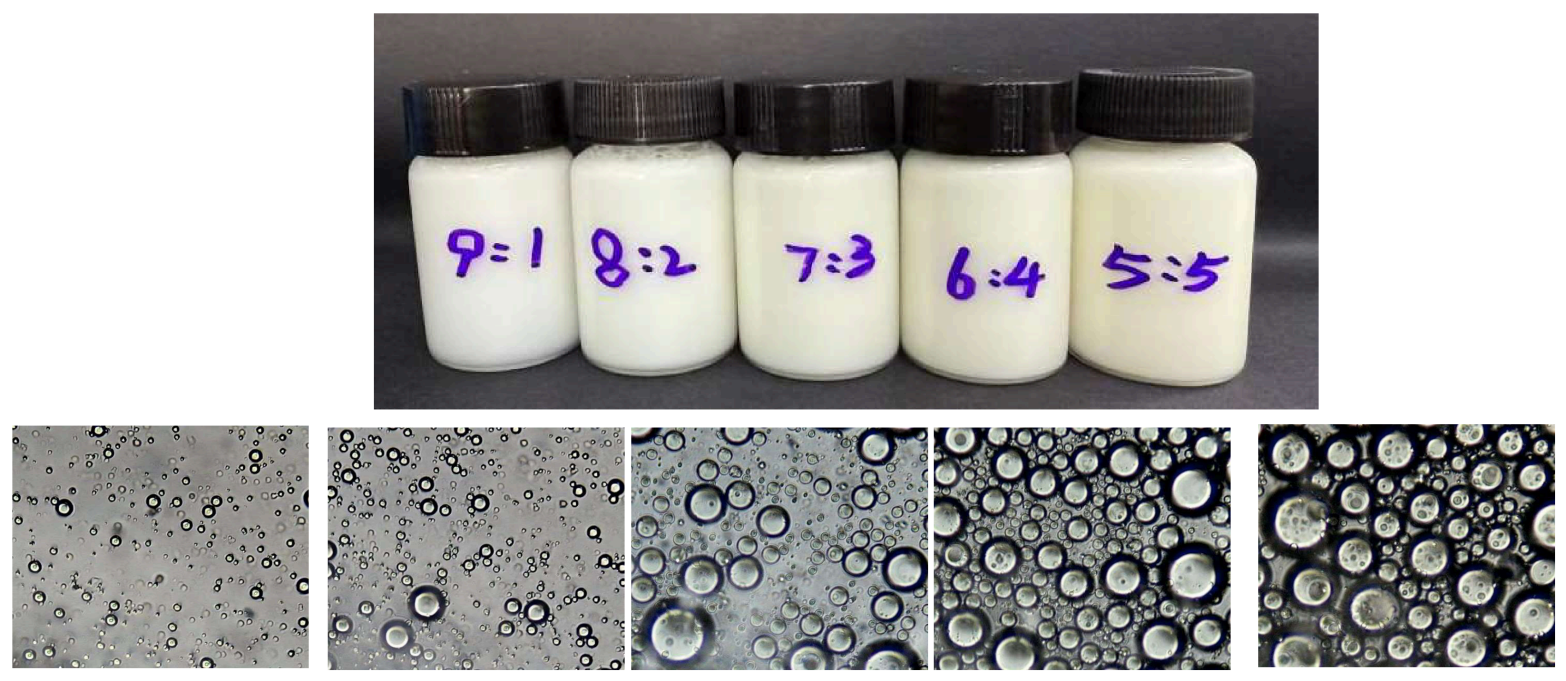
Macroscopic appearance and microscopic morphology of emulsions stabilized by PGEs. The handwritten content in the figure indicates different water-to-oil phase ratios.

The effects of PGEs on the emulsion, at both the macroscopic and microscopic scales, are presented in Figure 3. As the oil fraction increased, the volume of oil that could be encapsulated by per unit mass of PGEs continuously rose, which promoted droplet coalescence and growth [15]. At low oil fractions, sufficient PGE molecules could fully wrap oil droplets and form fine and homogeneous dispersed phases. Nevertheless, when the oil proportion was elevated, inadequate emulsifier molecules failed to form complete interfacial films around all oil droplets. Partially uncovered droplets tended to collide and coalesce, leading to a gradual increase in droplet size.

**Figure 4:**
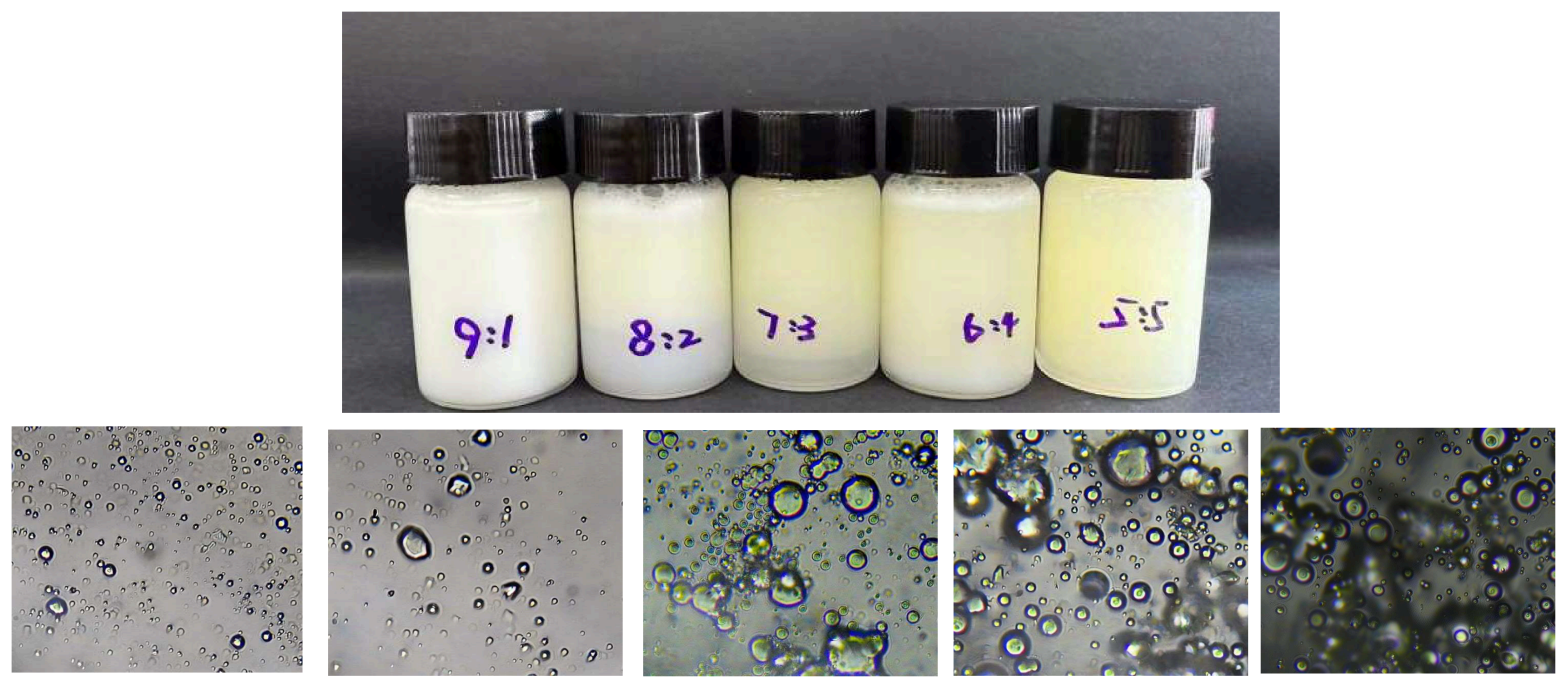
Macroscopic appearance and microstructures of emulsions stabilized by DATEM. The handwritten content in the figure indicates different water-to-oil phase ratios.

Figure 4 presents the macroscopic and microscopic morphologies of oil-in-water emulsions stabilized by DATEM. Macroscopically, obvious stratification and oil–water separation were observed in all groups except the 9:1 water–oil ratio group. Combined with the microscopic results, only the low-oil-fraction group contained tiny oil droplets [16]. As the oil fraction increased, a high number of large, densely packed and interconnected droplets emerged, forming visible flocculent aggregates that could be distinguished by naked eyes.

#### 3.1.3. Comparison of Droplet Size and PDI of Emulsions Stabilized by Three Emulsifiers

**Figure 5:**
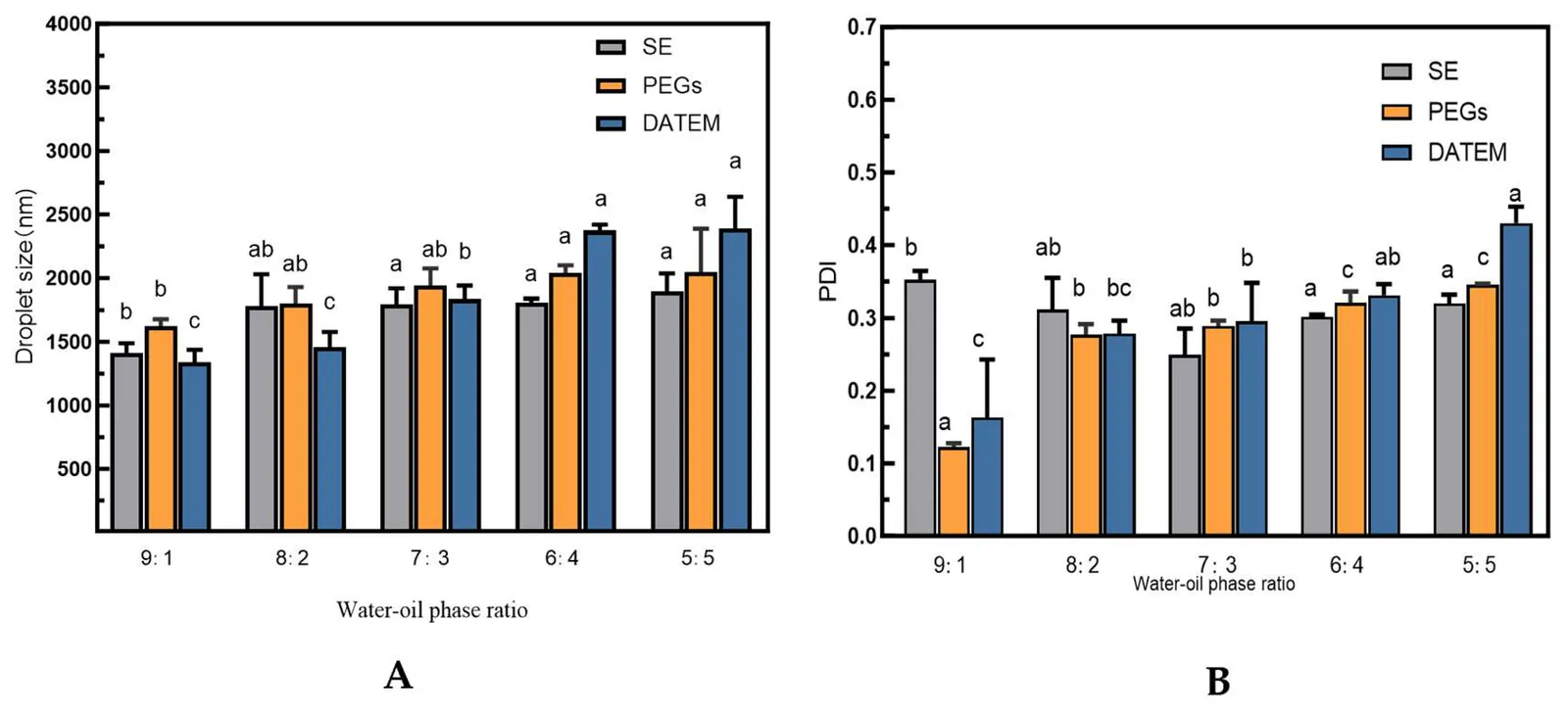
Droplet size and PDI of emulsions stabilized by three emulsifiers. (**A**) Droplet size; (**B**) PDI. Different lowercase letters above the bars denote significant differences between groups (*p* < 0.05, one-way ANOVA); bars sharing the same letter are not statistically different.

As illustrated in Figure 5A, as the water fraction decreased and the oil fraction increased gradually, the droplet sizes of emulsions stabilized by SE and PGEs remained at relatively low levels without drastic increases throughout all treatments, indicating that these two emulsifiers could effectively refine oil droplets even under high oil fraction conditions. In contrast, the droplet size of DATEM-stabilized emulsions rose significantly with the increase in oil proportion, reaching the maximum value at the water–oil ratio of 5:5 among all samples, which demonstrated that DATEM possessed the weakest capacity to maintain tiny droplets in high-oil systems.

According to the PDI results displayed in Figure 5B, the PDI value of PGEs was the lowest at the water–oil ratio of 9:1 (low oil fraction), suggesting the most uniform droplet size and optimal dispersion homogeneity. This result was consistent with the research of Zheng et al. [17]. With the continuous elevation in the oil fraction, the PDI values of all three emulsion groups increased moderately. Among them, DATEM exhibited the most remarkable growth in PDI and obtained the highest PDI at the 5:5 water–oil ratio. This revealed an extremely broad droplet size distribution and severely impaired homogeneity of the DATEM emulsion system under high oil loading.

#### 3.1.4. Light Transmittance of Emulsions

**Figure 6:**
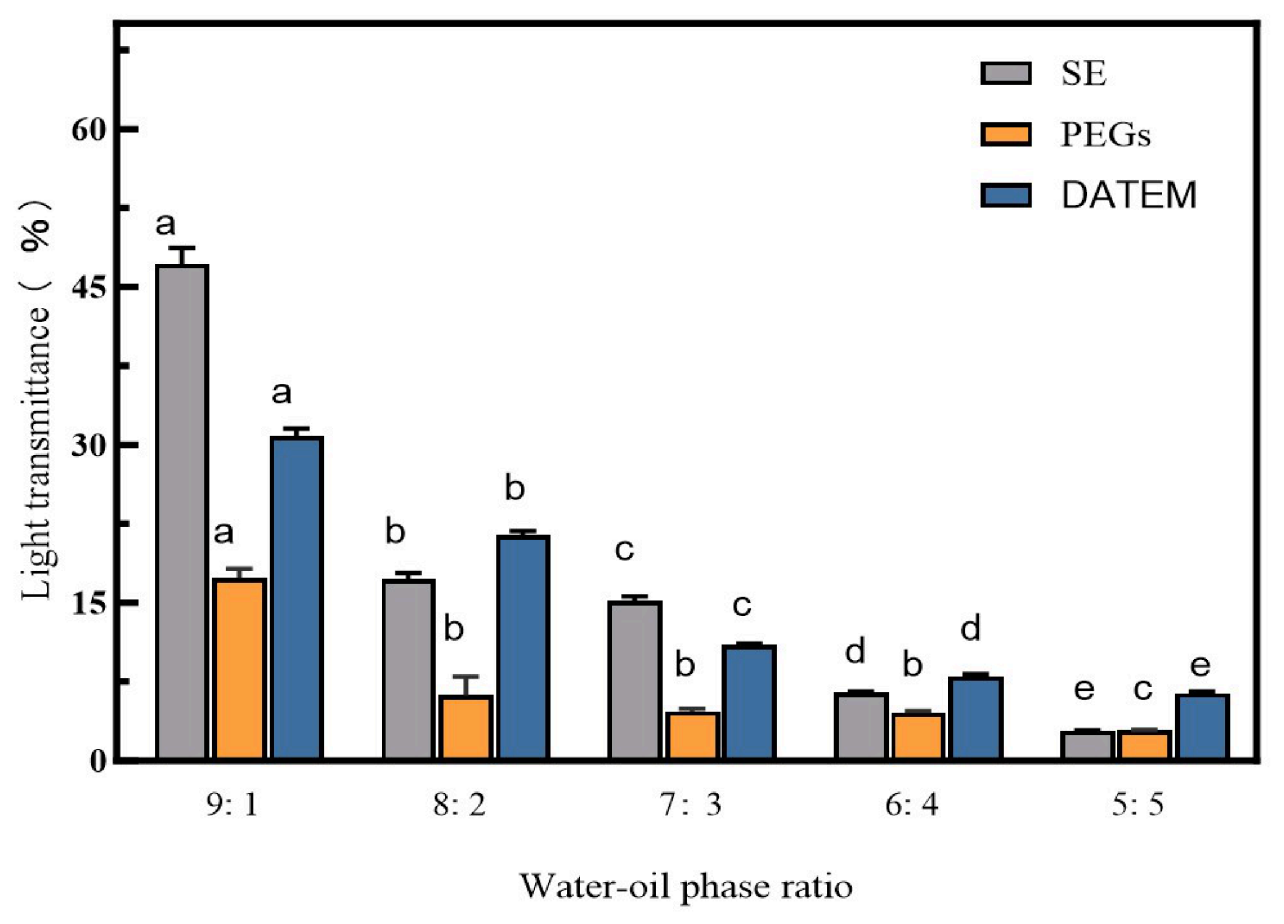
Comparison of light transmittance of emulsions. Different lowercase letters above the bars denote significant differences between groups (*p* < 0.05, one-way ANOVA); bars sharing the same letter are not statistically different.

Figure 6 illustrates the variation in light transmittance of emulsions stabilized by three emulsifiers across different water–oil ratios. The transmittance values of all emulsion groups continuously decreased as the water–oil ratio shifted from 9:1 to 5:5. This trend could be explained by the increased quantity and density of dispersed oil droplets with rising oil fraction. More light was scattered and blocked when passing through the emulsion system, thereby reducing light transmittance.

From the perspective of the light scattering theory, Amadei et al. [18] clarified the correlation between droplet size and emulsion transmittance: emulsions tend to be more transparent when oil droplets are much smaller than the wavelength of incident light. This conclusion agrees well with the present experimental results. Combined with the droplet size data above, the gradual growth of oil droplets at higher oil fractions ultimately led to the reduction in light transmittance.

#### 3.1.5. CI of Emulsions During 20 Days of Storage

**Figure 7:**
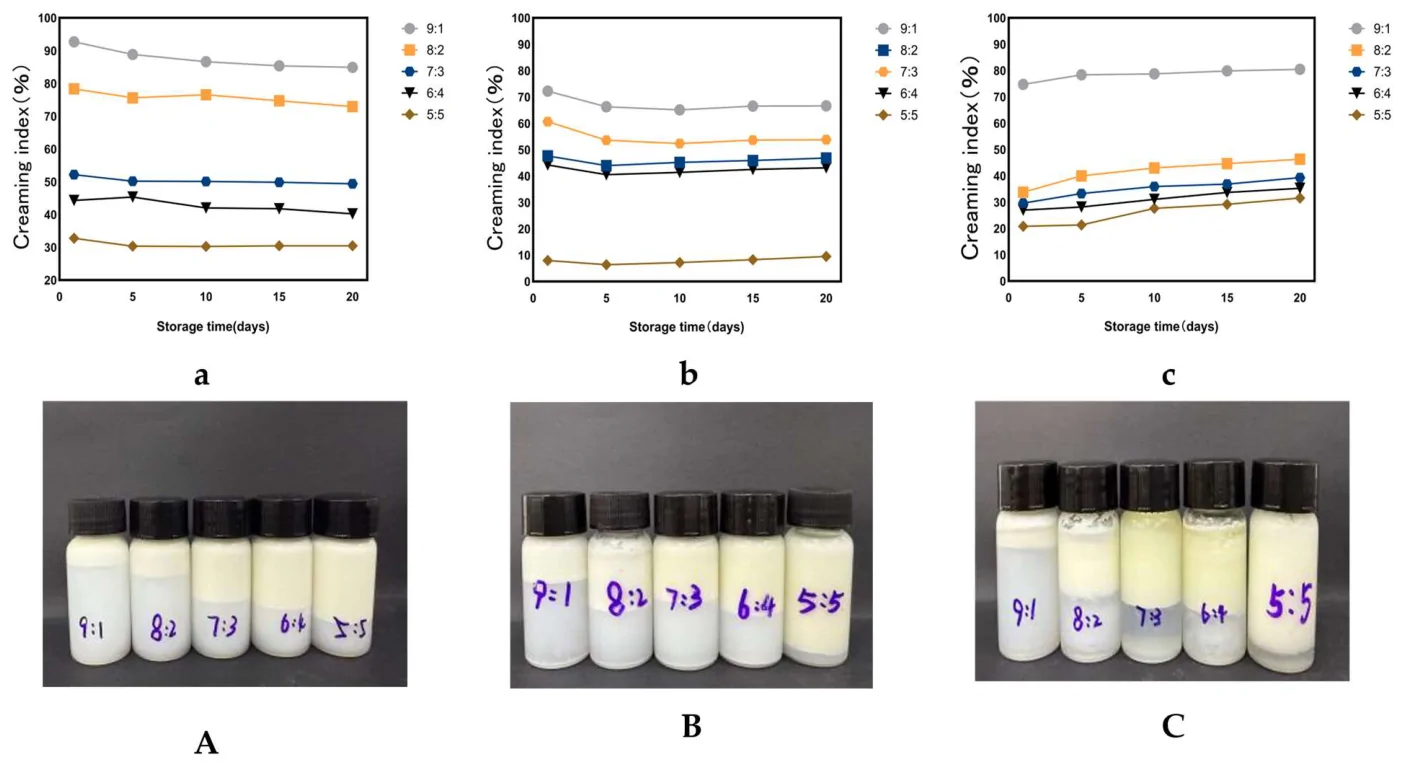
(**a**–**c**) Comparison of creaming index (20-day storage stability) of emulsions stabilized by three emulsifiers. (**a**) the SE group, (**b**) the PGEs group, (**c**) the DATEM group. (**A**–**C**) Visual appearance of emulsions stabilized by three emulsifiers after 20 days of storage. (**A**) the SE group, (**B**) the PGEs group, (**C**) the DATEM group. The handwritten content in the figure indicates different water-to-oil phase ratios.

The variations in CI of emulsions with different water–oil ratios during the 20-day storage period are shown in Figure 7a–c, while the appearance morphologies of the emulsions after 20 days of storage are shown in Figure 7A–C. Emulsions stabilized by SE and PGEs exhibited a consistent trend: the creaming index gradually decreased with the increase in oil fraction, indicating an obvious improvement in storage resistance against creaming. By contrast, the CI values of all DATEM emulsions rose slowly over storage time, which implied an increasing risk of phase separation during long-term standing. Among all DATEM groups, the sample at the water–oil ratio of 9:1 possessed the maximum CI value with the highest creaming risk, while the 5:5 group had the lowest CI and the optimal anti-creaming performance.

### 3.2. Effects of Different SE Dosages on Emulsion Properties

#### 3.2.1. Effects of SE Dosage on ESI

**Figure 8:**
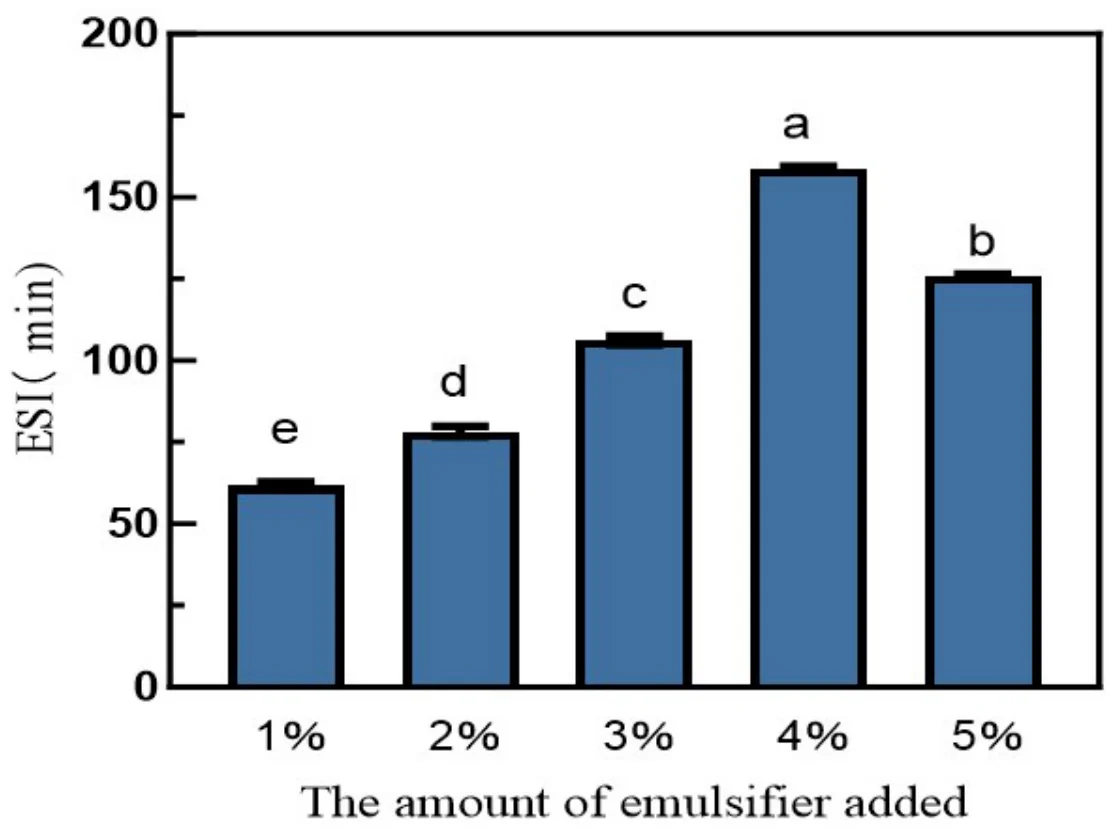
ESI of emulsions prepared with different SE dosages. Different lowercase letters above the bars denote significant differences between groups (*p* < 0.05, one-way ANOVA); bars sharing the same letter are not statistically different.

The effect of SE dosage on ESI is presented in Figure 8. As the SE dosage increased gradually from 1% to 4%, the ESI value continuously rose from approximately 60 to a peak of 160. Within this range, sufficient SE molecules arranged densely at the oil–water interface to form compact and robust interfacial films, effectively enhancing the anti-aggregation capacity of oil droplets. Nevertheless, a remarkable decline in the ESI was observed when the SE dosage was further increased to 5%. Excess emulsifier molecules failed to adsorb onto the oil–water interface and tended to aggregate within the continuous phase, disrupting the original interfacial balance and deteriorating overall emulsifying activity.

Under the experimental conditions of this study, 4% was identified as the optimal threshold dosage of SE for the emulsion system, which achieved the best emulsion stability while controlling material costs. Similar observations have been reported by Fu et al. [19].

#### 3.2.2. Effects of SE Dosage on Macroscopic Appearance and Microscopic Morphology of Emulsions

**Figure 9:**
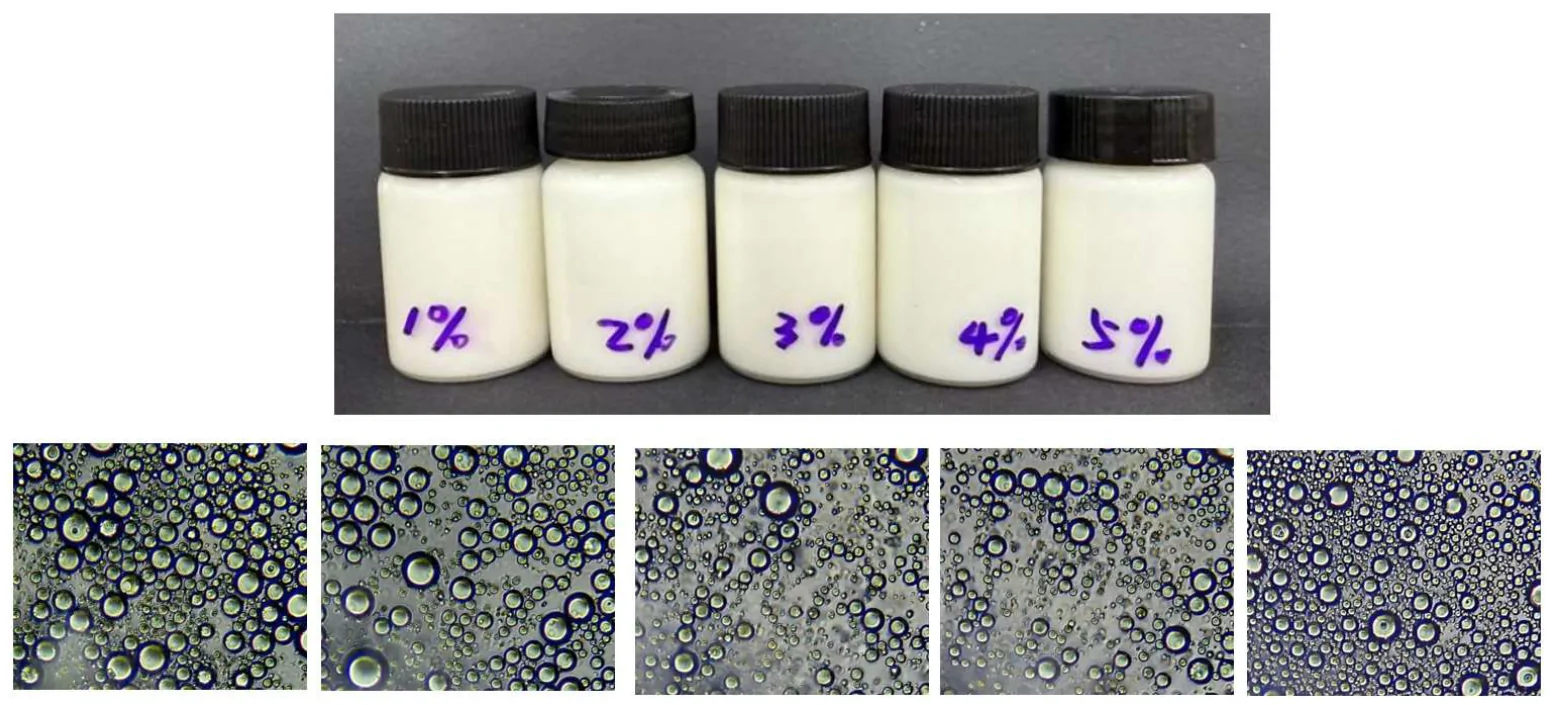
Macroscopic appearance and microscopic morphology of emulsions with different SE dosages. The handwritten content in the figure indicates different addition levels of SE.

The macroscopic and microscopic effects of SE addition levels on the emulsion are shown in Figure 9. All five groups of emulsions with different SE dosages exhibited a uniform milky-white liquid appearance without obvious phase separation, oil creaming, flocculation or sedimentation, maintaining favorable macroscopic homogeneity. This result revealed that the emulsion system remained macroscopically stable without severe demulsification within the SE dosage range of 1–5%.

Optical microscopic observations of dispersed oil droplets displayed gradient variations in droplet size and distribution. At the SE dosage of 1%, the average droplet size was relatively large with abundant big droplets and fewer total droplets in the field of view. Insufficient emulsifier molecules could not fully cover all newly formed oil droplets at this dosage. As the SE dosage increased from 2% to 5%, more adsorption sites became available to encapsulate oil droplets, and abundant emulsifier molecules rapidly adsorbed onto the oil–water interface. Correspondingly, the average droplet size gradually decreased, accompanied by an elevated proportion of small droplets. Droplets were densely and uniformly distributed, leading to a narrower droplet size distribution and forming emulsions with excellent dispersity and homogeneous microscopic morphology.

#### 3.2.3. PDI of Emulsions with Different SE Dosages

**Figure 10:**
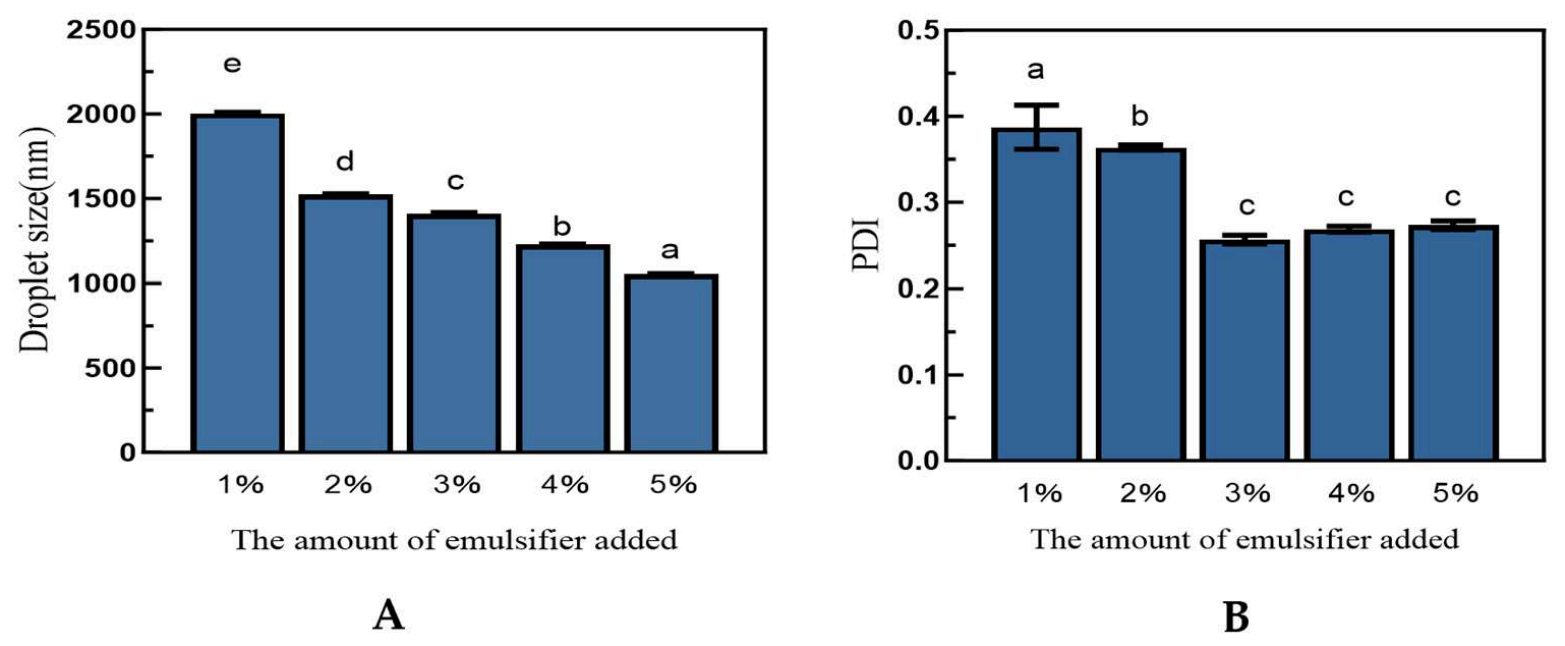
(**A**) Droplet size of SE-stabilized emulsions at different SE dosages. (**B**) PDI of SE-stabilized emulsions at different SE dosages. Different lowercase letters above the bars denote significant differences between groups (*p* < 0.05, one-way ANOVA); bars sharing the same letter are not statistically different.

With fixed oil fraction and identical emulsification processing parameters, the SE dosage was adjusted from 1% to 5%, and the droplet size and PDI of the resultant emulsions were characterized, as presented in Figure 10A,B.

Variation in droplet size: At the SE dosage of 1%, the insufficient emulsifier molecules failed to fully cover the interface of newly formed oil droplets, which greatly increased the coalescence probability of small droplets, resulting in the maximum average droplet size. As the SE dosage increased from 1% to 5%, abundant emulsifier molecules rapidly coated oil droplets during homogenization and sharply reduced interfacial tension, fundamentally inhibiting droplet coalescence and gradually decreasing the average droplet size of emulsions.

Variation in PDI: The changing trend of the PDI was highly consistent with that of the droplet size. The emulsion with 1% SE exhibited the highest PDI, indicating an extremely broad droplet size distribution. The dispersion uniformity of droplets was continuously optimized with an increasing SE dosage, and the difference in the proportion of droplets with various sizes was narrowed, accompanied by a gradual decline in the PDI. Low PDI values were maintained within the dosage range of 3–5%, demonstrating a significantly narrowed droplet size distribution and superior homogeneity of the emulsion system.

#### 3.2.4. Effects of SE Dosage on the CI (20-Day Storage Stability) of Emulsions

**Figure 11:**
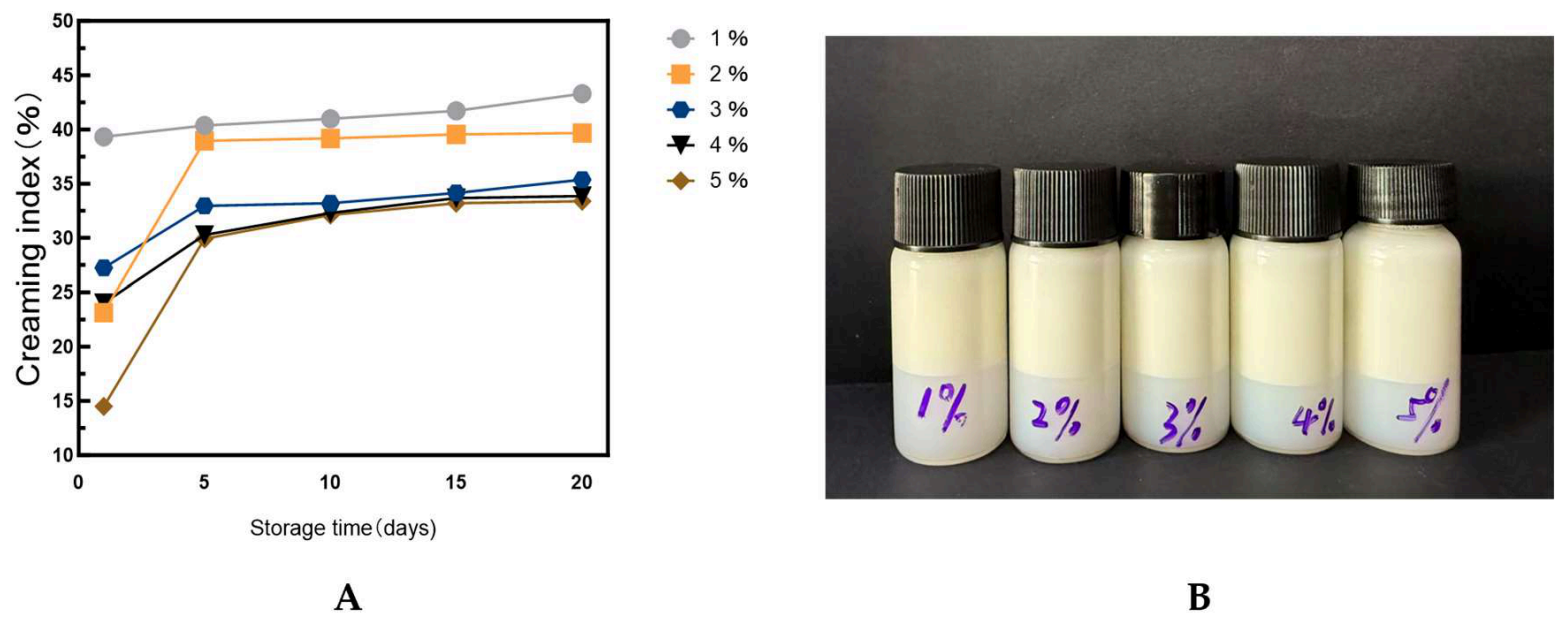
Effects of different emulsifier addition levels on the storage stability of emulsions. (**A**) The effects of varying SE dosages on the CI of SE-stabilized emulsions. (**B**) Effect of different addition levels of SE on the appearance morphology of emulsions. The handwritten content in the figure indicates different addition levels of SE.

The effects of varying SE dosages on the CI of SE-stabilized emulsions are illustrated in Figure 11A, while their macroscopic visual appearances are presented in Figure 11B. A gradual upward trend in CI values was observed for all sample groups with extended storage time, indicating that creaming and phase separation occurred to varying degrees in all emulsions during static storage. Nevertheless, remarkable discrepancies were detected in both creaming rates and final CI values among formulations with different SE dosages.

For the 1% SE group, CI values remained the highest across the entire storage period, rising progressively from an initial value of 39% to approximately 43% on day 20. This group exhibited the most severe creaming behavior and the poorest colloidal stability.

The 2% SE sample maintained CI values within the range of 23–39%, substantially lower than the 1% SE group. Furthermore, the CI barely increased after 5 days of storage, delivering superior overall stability relative to the 1% SE formulation.

Emulsions stabilized with 3% and 4% SE displayed comparable trends in CI variation, with final CI values falling between 33% and 35% on day 20; their storage stability was notably enhanced compared with the two lower-dosage groups.

The 5% SE group possessed the lowest initial CI at roughly 14%. The CI climbed rapidly to nearly 30% within the first 5 days, followed by a mild slow increase thereafter. Overall, this group maintained relatively low CI levels throughout the full 20-day storage cycle.

Formulation screening experiments confirmed that SE acted as the optimal emulsifier, with the optimal water–oil mass ratio determined as 6:4 and the optimal SE dosage set at 4% (*w*/*w*, based on oil mass). Emulsions prepared under these conditions exhibited the maximum emulsifying stability. Accordingly, crude emulsions were fabricated with the above optimized formulation prior to subsequent high-pressure microfluidization treatment.

Ghasemi et al. [20] also confirmed that increasing the emulsifier dosage could effectively inhibit droplet coalescence, yielding highly stable emulsions with smaller droplet sizes and narrower size distributions.

### 3.3. Effects of High-Pressure Microfluidization Pressure on Emulsion Properties

#### 3.3.1. Effects of High-Pressure Microfluidization Pressure on ESI

**Figure 12:**
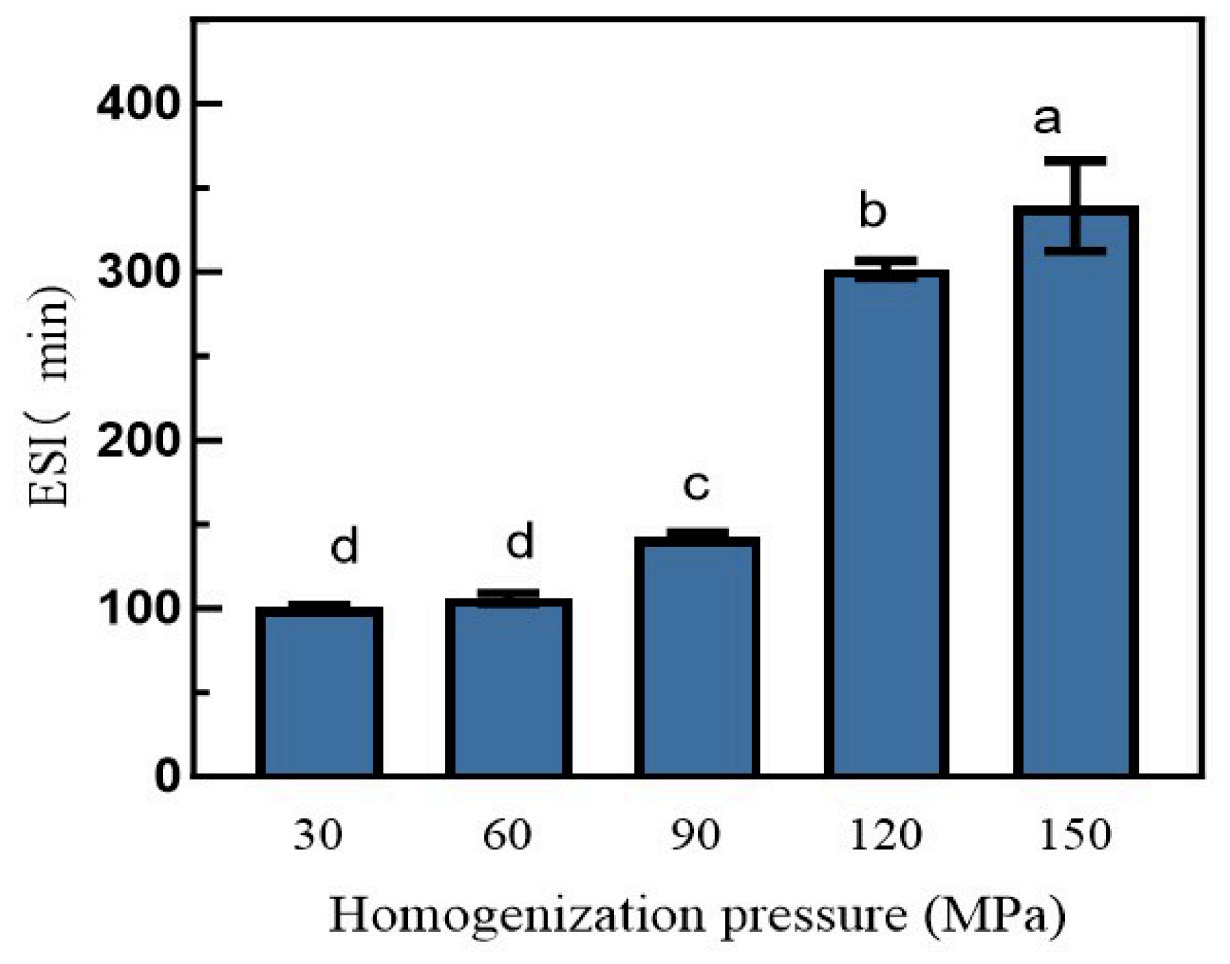
ESI of SE-stabilized emulsions treated at different microfluidization pressures. Different lowercase letters above the bars denote significant differences between groups (*p* < 0.05, one-way ANOVA); bars sharing the same letter are not statistically different.

The outstanding emulsifying activity of emulsions treated by microfluidization can be attributed to the combined forces generated by ultrahigh pressure, high-velocity impingement, cavitation, and intense shear rate [21]. The impacts of various microfluidization pressures on the ESI are presented in Figure 12. The ESI value exhibited a consistent monotonic upward trend with the elevation of processing pressure. At 30 MPa, the ESI was merely around 100 min, representing a relatively low emulsifying level.

As the pressure gradually increased to 60 MPa and then 90 MPa, the ESI rose steadily from 100 min to approximately 140 min. A dramatic surge in the ESI was observed when the pressure further increased from 90 MPa to 120 MPa, where the ESI exceeded 300 min, accompanied by a markedly amplified improvement in emulsification. The maximum ESI value across all tested samples was achieved at 150 MPa, reaching nearly 340 min, which was more than three times that of emulsions processed at 30 MPa.

#### 3.3.2. Effects of Microfluidization Pressure on CLSM Images of Emulsions

The structural characteristics and component distribution of food matrices are critical for elucidating their physicomechanical, chemical, thermal and biological properties, which directly determine food quality, safety and consumer acceptability. CLSM has emerged as an effective technique to characterize micro- and nano-scale biopolymer matrix architectures and component distribution [22].

**Figure 13:**
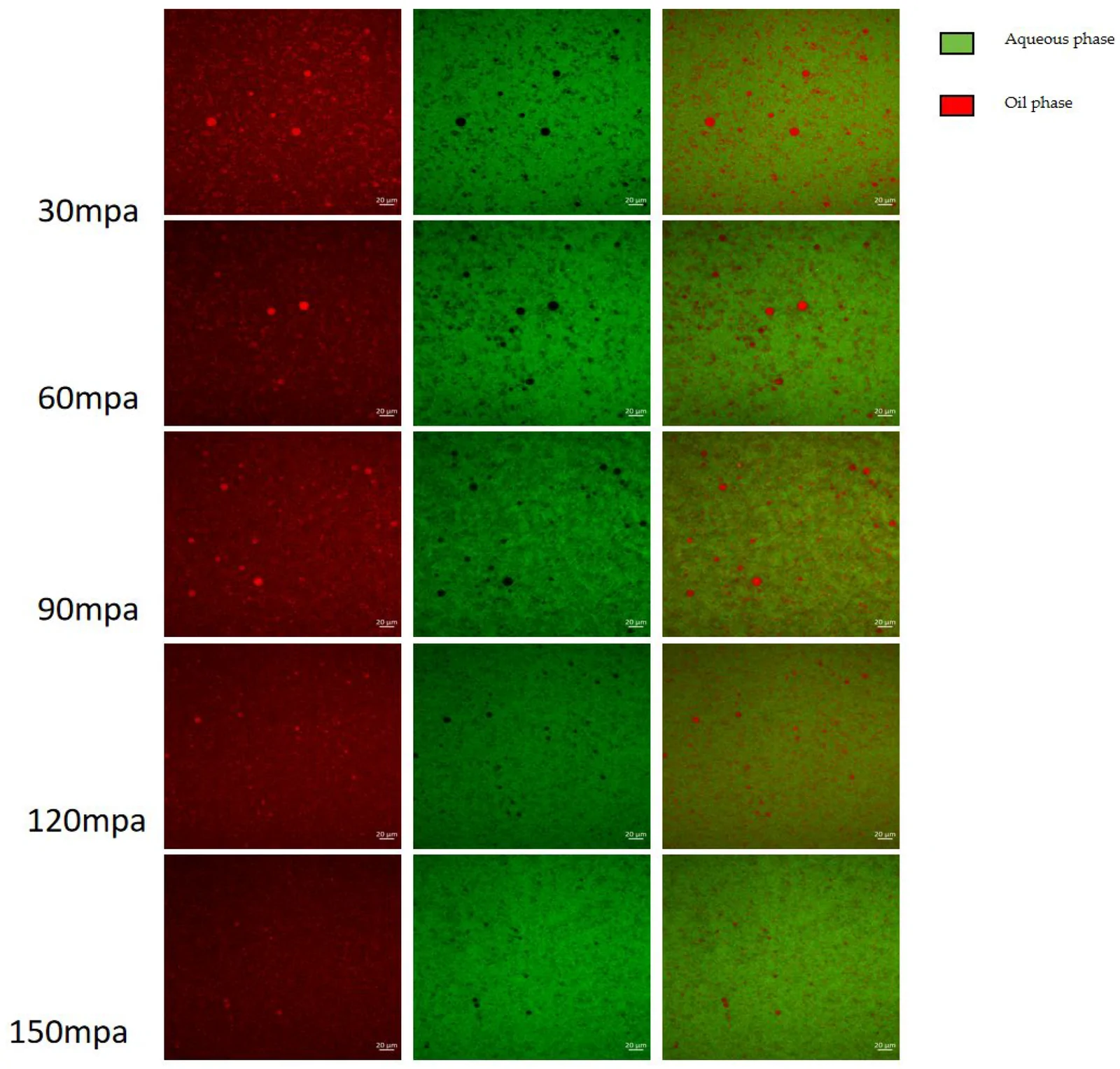
CLSM micrographs of SE-stabilized emulsions treated at different microfluidization pressures (emulsions stained with Nile Blue and Nile Red. Green fluorescence indicates the aqueous phase (stained with Nile Blue), and red fluorescence indicates the oil phase (stained with Nile Red). The scale bar in the figure represents 20 µm.

As illustrated in Figure 13, all five microfluidized emulsion groups exhibited homogeneous and stable dispersion. Even the sample treated at a low pressure of 30 MPa did not display severe destabilization accompanied by abundant free large oil droplets, and regular structural variations were detected along the pressure gradient. As the microfluidization pressure progressively increased from 30 MPa to 150 MPa, the walnut oil droplets observed via CLSM displayed a gradual and stepwise reduction in size, alongside more uniform and compact droplet distribution. Meanwhile, the emulsifier molecules formed more continuous and intact interfacial layers covering oil droplets with elevated pressure and generated tighter interconnections with the fructooligosaccharide aqueous network. Consequently, highly homogeneous microstructures with negligible differences in droplet dimension were obtained within the pressure range of 120–150 MPa.

#### 3.3.3. Effects of Microfluidization Pressure on Macroscopic Appearance and Microscopic Morphology of Emulsions

**Figure 14:**
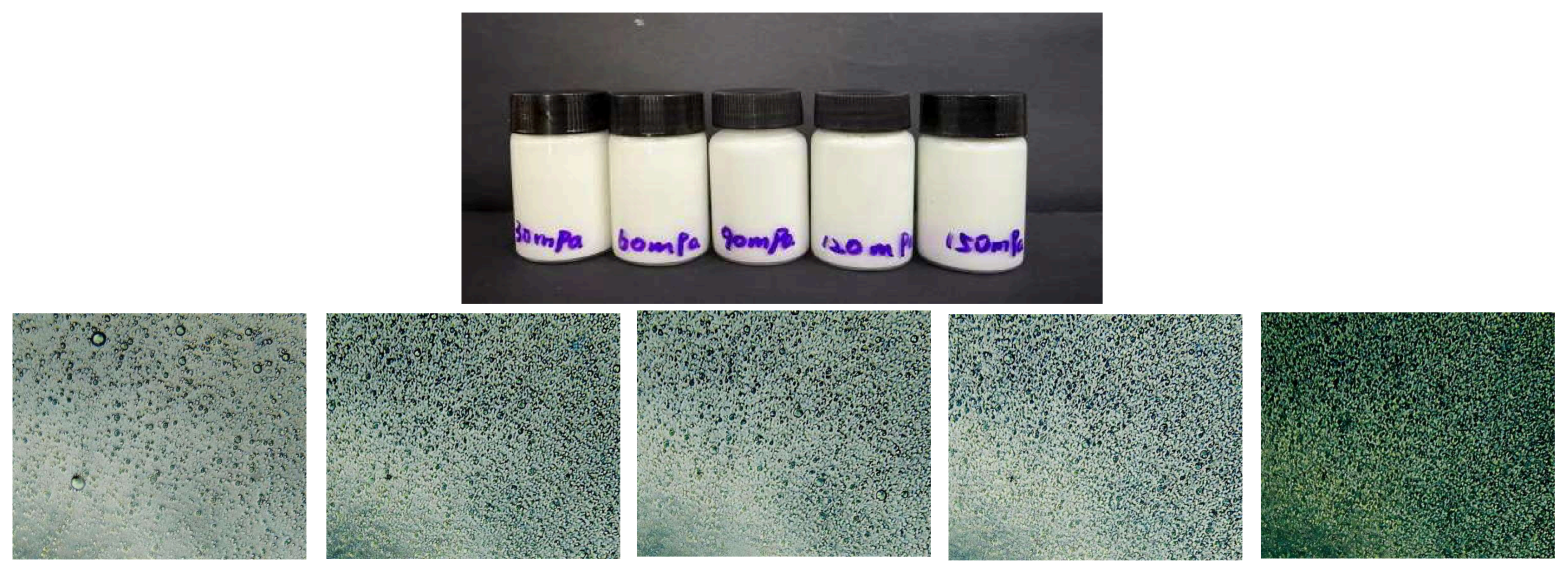
Macroscopic appearance and microscopic morphology of SE-stabilized emulsions treated at different microfluidization pressures. The handwritten content in the figure indicates different homogenization pressures used for treating the emulsions.

HPM remarkably modulates the particle characteristics of emulsions. Increasing processing pressure leads to progressive droplet size reduction and improved homogeneity of droplet size distribution [23]. In Figure 14, the five samples from left to right correspond to microfluidization pressures of 30 MPa, 60 MPa, 90 MPa, 120 MPa and 150 MPa, respectively. All formulations exhibited a uniform milky-white liquid appearance without obvious phase separation, free oil or creaming-induced destabilization. This finding indicated that microfluidization treatment at the lowest tested pressure of 30 MPa could already construct a fundamentally stable dispersed emulsion system.

The five microscopic micrographs at the bottom correspond to the pressure groups above and presented a distinct gradient structural evolution. Within the pressure range of 30–150 MPa, oversized abnormal droplets were nearly eliminated from the field of view, accompanied by a prominent decline in overall droplet size and a remarkable increase in droplet packing density. A dense microstructure with negligible differences in droplet dimension was ultimately generated, reflecting continuously optimized emulsification performance. These observations were highly consistent with the previous CLSM characterization results.

#### 3.3.4. Effects of Microfluidization Pressure on Droplet Size, PDI and Zeta Potential of Emulsions

**Figure 15:**
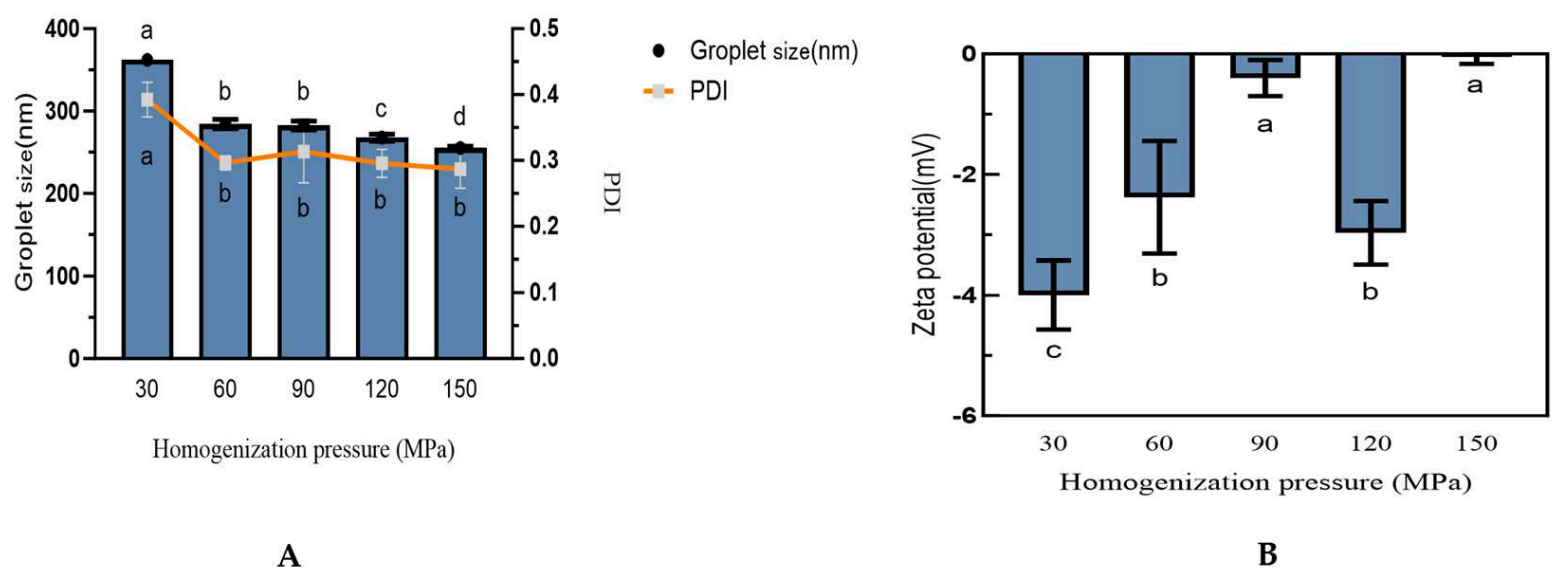
(**A**) Droplet size and PDI of SE-stabilized emulsions under varying microfluidization pressures. (**B**) Zeta potential of SE-stabilized emulsions treated with different microfluidization pressures. Different lowercase letters above the bars denote significant differences between groups (*p* < 0.05, one-way ANOVA); bars sharing the same letter are not statistically different.

Droplet size acts as a critical determinant of emulsion functional performance [24]. All emulsions fabricated in the present study exhibited average droplet diameters below 400 nm. Consistent with the CLSM observations, high-pressure microfluidization homogenization significantly reduced droplet size. As the processing pressure increased, the average droplet size gradually decreased from 362 nm (30 MPa) to 284.17 nm (60 MPa), 282.47 nm (90 MPa), 267.7 nm (120 MPa) and 255.07 nm (150 MPa), demonstrating that microfluidization exerts a remarkable refining effect on emulsion droplet dimensions. This homogenization technique effectively narrowed the droplet size distribution, with the PDI value declining from 0.392 to 0.287, as displayed in Figure 15A. Such a trend could be attributed to intense high-speed shear and cavitation effects generated during microfluidization, which reorganize droplet populations and improve the homogeneity of the emulsion system [25].

The regulatory effects of homogenization pressure on zeta potential were further investigated, and all tested samples exhibited negative zeta potential values (Figure 15B), revealing that oil droplets carried net negative surface charges. Notably, the absolute zeta potential values of all formulations were substantially lower than 30 mV, implying that electrostatic repulsion alone could not confer outstanding colloidal stability to the system. This phenomenon stemmed from the nonionic nature of the SE emulsifier [26]. Instead, the long-term storage stability of the developed emulsions was mainly governed by the oriented adsorption of sucrose ester molecules at the oil–water interface [27]. The obtained variation trends of the zeta potential provide clear experimental support for further optimizing the physical stability of walnut oil–fructooligosaccharide emulsions via adjusting homogenization parameters and emulsifier dosage in subsequent research.

#### 3.3.5. Effects of Microfluidization Pressure on the Antioxidant Capacity of Emulsions

**Figure 16:**
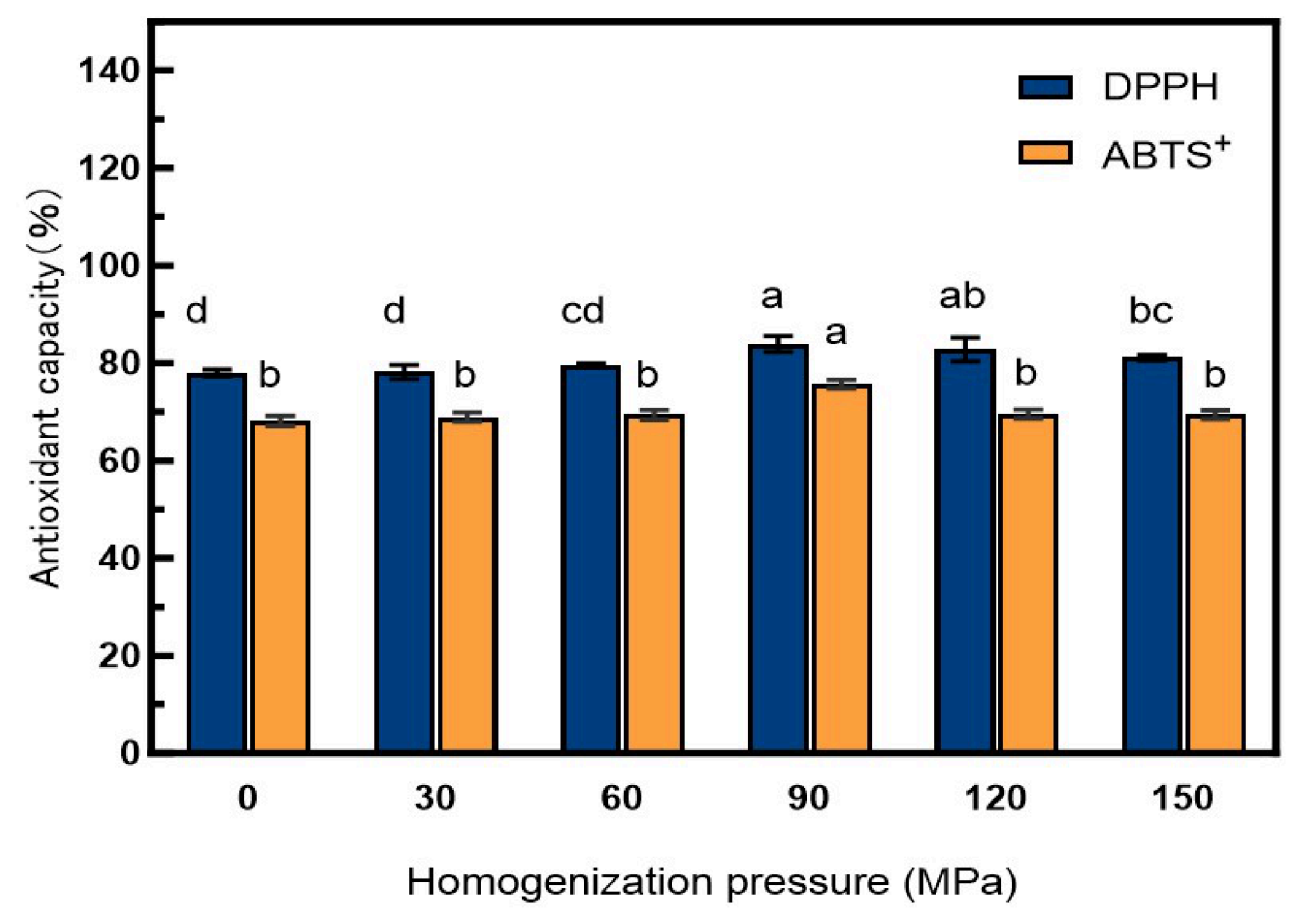
Effects of microfluidization pressure on antioxidant capacity of emulsions. Different lowercase letters above the bars denote significant differences between groups (*p* < 0.05, one-way ANOVA); bars sharing the same letter are not statistically different.

As shown in Figure 16, within the pressure range of 0–150 MPa, the antioxidant activity determined by the DPPH assay slightly fluctuated and increased initially, followed by a mild decline. The maximum antioxidant capacity was obtained at 90 MPa, with only marginal reductions observed at 120 MPa and 150 MPa. This result suggested that an excessively high microfluidization pressure could degrade antioxidant components [28]. In comparison, the ABTS assay exhibited much milder fluctuations throughout the tested pressure range, with values maintained at a comparable level and only a subtle minor peak occurring near 90 MPa.

#### 3.3.6. Effects of Microfluidization Pressure on the 20-Day Storage Stability of Emulsions

**Figure 17:**
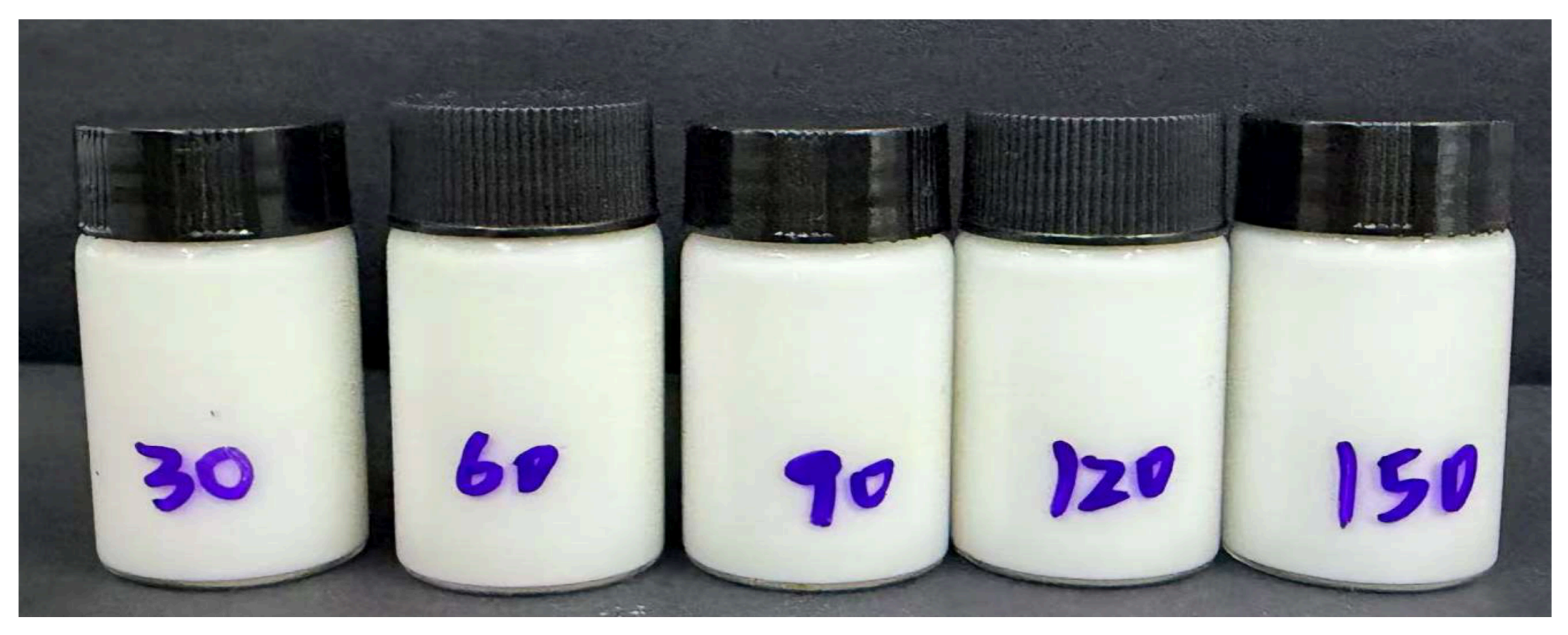
Macroscopic appearance of emulsions treated at different microfluidization pressures after 20 days of storage. The handwritten content in the figure indicates different homogenization pressures used for treating the emulsions.

All walnut oil–fructooligosaccharide emulsions fabricated by high-pressure microfluidization at 30, 60, 90, 120 and 150 MPa remained homogeneous milky-white dispersions after 20 days of storage under ambient temperature conditions. As shown in Figure 17, No phase separation, sedimentation at the bottom or free oil creaming on the top surface was observed for any treatment group, visually verifying that microfluidization homogenization greatly improves the long-term storage stability of the emulsion system.

As the homogenization pressure increased from 30 MPa to 150 MPa, the combined effects of high-speed shear, cavitation and impingement continuously refined walnut oil droplets and greatly improved the uniformity of droplet size distribution. Meanwhile, emulsifier molecules adsorbed and arranged more compactly and completely at the oil–water interface, which remarkably weakened the van der Waals attraction between droplets and further reinforced the steric stabilization effect. Macroscopically, the emulsions exhibited progressively improved visual homogeneity and showed no signs of destabilization during long-term storage.

Crude emulsions without high-pressure microfluidization treatment usually suffer from obvious creaming and free oil separation within a short storage period. In contrast, all microfluidized samples maintained uniform morphology over 20 days as shown in Figure 17, demonstrating that the intensive dispersion effect of ultrahigh-pressure microfluidization synergizes with the sucrose ester–fructooligosaccharide composite stabilization system to construct a long-term stable oil-in-water emulsion and effectively extend its storage shelf life.

### 3.4. Effects of Different Additives on Rice Quality

#### 3.4.1. Effects of Different Additives on Moisture Content of Cooked Rice

Distinct differences in the water loss rate were observed among the six sample groups. The boiled control group (Group 1) exhibited the highest water loss rate of nearly 56%, indicating that conventionally cooked rice suffered the fastest water migration and possessed the weakest water retention capacity. The water loss rate of the walnut oil group (Group 2) slightly decreased to approximately 52%, as single walnut oil addition could coat the starch structure on rice grain surfaces and retard the loss of free water. The fructooligosaccharide group (Group 3) showed a water loss rate of around 53%; the strong hydrophilicity of fructooligosaccharides enables them to bind to abundant water molecules and effectively improve the water holding capacity of cooked rice. The walnut oil–fructooligosaccharide direct physical blend group (Group 4, without emulsifier addition and without homogenization, with the ingredients directly added before cooking) exhibited a water loss rate of around 52%, indicating that simple physical mixing of the ingredients could also maintain, to a certain degree, the water retention properties of the rice. The crude emulsion group (Group 5) displayed a water loss rate of about 52%, which also maintained favorable water retention performance. Notably, the emulsion prepared at 150 MPa (Group 6) achieved the lowest water loss rate of 51% across all treatments. This result reveals that the combination of walnut oil and fructooligosaccharide followed by high-pressure microfluidization treatment retained outstanding water retention properties. This phenomenon could be explained by the emulsion restricting starch granule swelling and water mobility, thereby regulating the water holding capacity of rice starch [29] and further modulating the water retention behavior of cooked rice.

**Figure 18:**
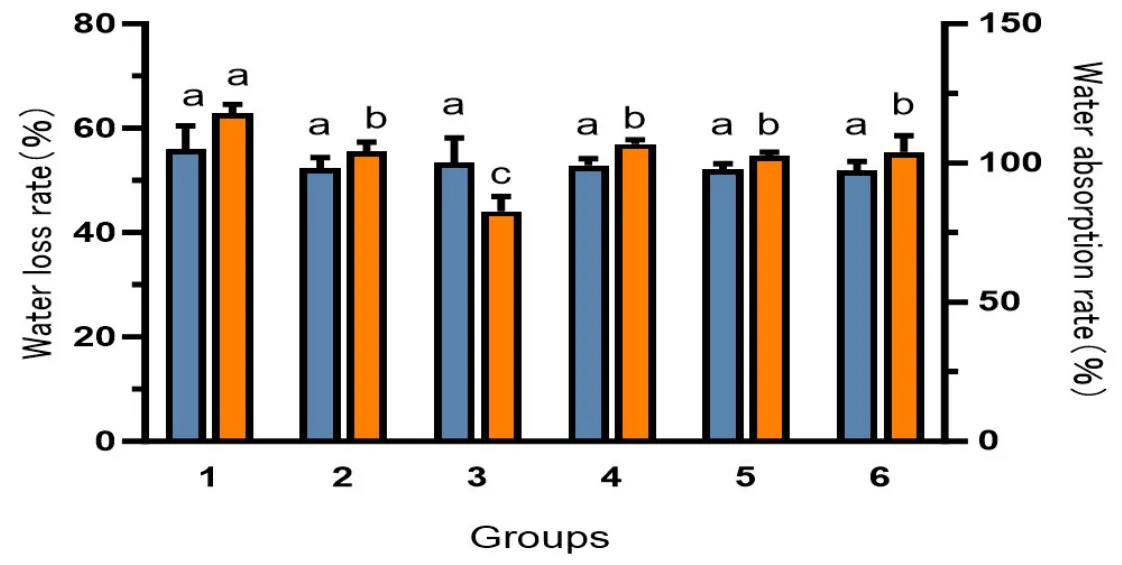
Moisture-related indicators of cooked rice supplemented with different additives. Different lowercase letters above the bars denote significant differences between groups (*p* < 0.05, one-way ANOVA); bars sharing the same letter are not statistically different.

An excessively high water absorption rate leads to overly sticky rice, while insufficient water absorption results in a hard texture. As illustrated in Figure 18, all five additive treatments reduced the water absorption rate of cooked rice. The fructooligosaccharide group had the smallest water absorption rate at 82.62%, whereas the control group possessed the highest value of 118.01%. Consequently, these two groups presented undesirable textural defects of excessively hard or sticky mouthfeel. By contrast, rice treated with walnut oil, a simple physical mixture of the walnut oil and FOS, crude emulsion, and 150 MPa of microfluidized emulsion exhibited moderate texture with higher sensory acceptability.

#### 3.4.2. Effects of Different Additives on the Color Attributes of Cooked Rice

The effects of different additives on the color of cooked rice are shown in Table 1. Obvious gradient differences in the L* value (lightness) were observed among the six cooked rice groups. The control boiled group and fructooligosaccharide (FOS) group exhibited nearly identical L* values at approximately 68, representing the lowest lightness across all treatments, which was consistent with the whiteness of conventionally cooked white rice. The walnut oil and FOS combined group showed a slightly elevated L* value of 69.93, which was marginally higher than the two lowest-lightness groups, indicating that the coexistence of walnut oil and FOS produced a mild synergistic effect on the surface brightness of cooked rice without reaching the lightness level of the pure walnut oil group. The crude emulsion group had an L* value of 70.37, showing slightly higher lightness than the control group and an intermediate brightness between plain boiled rice and rice cooked with pure walnut oil. The L* value of the walnut oil group increased to 73.9, which could be attributed to the high abundance of unsaturated fatty acids in walnut oil with superior fluidity [30]. These lipids evenly distribute on the surface and within the pores of rice grains, effectively improving the lightness of cooked rice. Rice treated with the microfluidized emulsion at 150 MPa achieved the maximum L* value of 79.62, which was the highest among all samples. The nano-scaled dispersed emulsion system greatly enhanced the glossy and translucent appearance of cooked rice. Consistent with the present findings, Long et al. [31] reported that flaxseed gum/egg white protein-based curcumin nano-emulsion coatings could retard the decrease in the whiteness of fried rice.

#### 3.4.3. Effects of Different Additives on Textural Properties of Cooked Rice

The effects of different additives on the texture of cooked rice are shown in Table 2. Textural parameters can reflect the gel network state and retrogradation degree of rice starch, and four indicators including hardness, adhesiveness, springiness and chewiness jointly characterize the eating mouthfeel of cooked rice [32], with distinct gradient differences observed across all treatment groups. The control group cooked with pure water exhibited the maximum hardness of 6502.44 g and an extremely low springiness value of only 0.195, far lower than all additive-supplemented groups, alongside a chewiness of 514.40 g. These results indicate that cooked rice without exogenous additives possessed a dry, rigid texture, poor resilience and high chewing resistance, resulting in a coarse eating quality.

Single walnut oil addition could coat starch granules via hydrophobic lipid molecules and reduce hydrogen bonding between starch chains [33], which remarkably decreased rice hardness to 4022.51 g and synchronously lowered adhesiveness to 761.94 g. Nevertheless, springiness was only slightly improved to 0.27, representing a limited textural modification effect. Although rice became softer, it lacked resilient and chewy mouthfeel. Fructooligosaccharide (FOS) alone effectively restricted water mobility within cooked rice [34], reducing hardness to 3595.43 g and drastically elevating springiness to 0.66, while adhesiveness slightly exceeded that of the control group.

Consequently, rice supplemented with pure FOS presented a soft texture accompanied by excessive stickiness on the palate.

The group with direct physical blending of walnut oil and fructooligosaccharides (FOS) exhibited a hardness of 3635.72 g, which was significantly lower than that of the group with walnut oil addition alone and comparable to that of the group with FOS addition alone. Its springiness further increased to 0.71, higher than that observed with FOS addition alone, indicating that the coexistence of oil and FOS already exerted a synergistic effect in modulating the starch gel network and enhancing the resilience of the cooked rice. The adhesiveness of this group was 1465.60 g·s, falling within the range of the water-only control and the FOS group, thus still retaining a relatively strong adhesive character. The chewiness was 495.16 g, showing no significant advantage over the individual addition groups. Collectively, these results suggest that simple physical mixing of the two components, while capable of simultaneously reducing hardness and increasing springiness, failed to effectively alleviate the tooth-packing issue induced by FOS. The inhomogeneous dispersion of the components indicated that the full synergistic potential of the combination had not yet been fully exploited.

When walnut oil and FOS were combined to prepare crude emulsion, synergistic effects occurred between lipid and carbohydrate fractions, further strengthening the softening effect. The hardness was reduced to 3387.27 g, springiness remained at 0.67, adhesiveness reached the peak value of 1614.10 g among all treatments, and chewiness declined to 435.37 g. Rice cooked with crude emulsion was soft and easy to chew, yet suffered from overly sticky texture.

After homogenization via high-pressure microfluidization at 150 MPa, emulsion droplets were drastically refined, allowing walnut oil and FOS to uniformly embed into the rice starch matrix. This treatment yielded the minimum hardness of 3297.22 g across all samples, which could be attributed to the formation of complexes between emulsifiers and starch molecules that soften rice texture [35]. Meanwhile, springiness reached the highest value of 0.74, conferring the optimal resilient and chewy property after mastication. Adhesiveness declined to 1172.03 g, which alleviated the excessive stickiness induced by FOS. Although the chewiness of 453.01 g was marginally higher than that of crude emulsion, it was still much lower than the control, single walnut oil and single FOS groups. The microfluidized emulsion balanced softness and a refreshing mouthfeel, overcame the textural drawbacks of single additives, and delivered the overall optimal texture performance.

In summary, single walnut oil only softened rice and reduced surface stickiness, without improving springiness or flavor; single FOS enhanced springiness and adhesiveness with limited softening capacity, and its flavor-improving effect was inferior to composite emulsions. Both single additives possessed obvious defects in mouthfeel and sensory quality. Although the simple walnut oil–FOS physical blend group concurrently lowered hardness and enhanced springiness, the adhesiveness remained appreciably high, indicating that the overall textural improvement was insufficient. The oil-in-water composite emulsion composed of walnut oil and FOS generated synergistic effects to simultaneously optimize the texture and flavor of cooked rice, outperforming separate supplementation of lipid or polysaccharide. Although non-homogenized crude emulsion greatly reduced chewing resistance, it suffered from excessive adhesiveness and uneven distribution of flavor compounds. High-pressure microfluidization at 150 MPa refined emulsion droplets to achieve uniform dispersion of walnut oil and FOS within rice starch molecules, maximally inhibiting starch retrogradation from the textural perspective, and producing high-quality cooked rice with low hardness, high springiness, moderate adhesiveness and easy chewiness.

#### 3.4.4. Effects of Different Additives on Volatile Flavor Compounds of Cooked Rice

**Figure 19:**
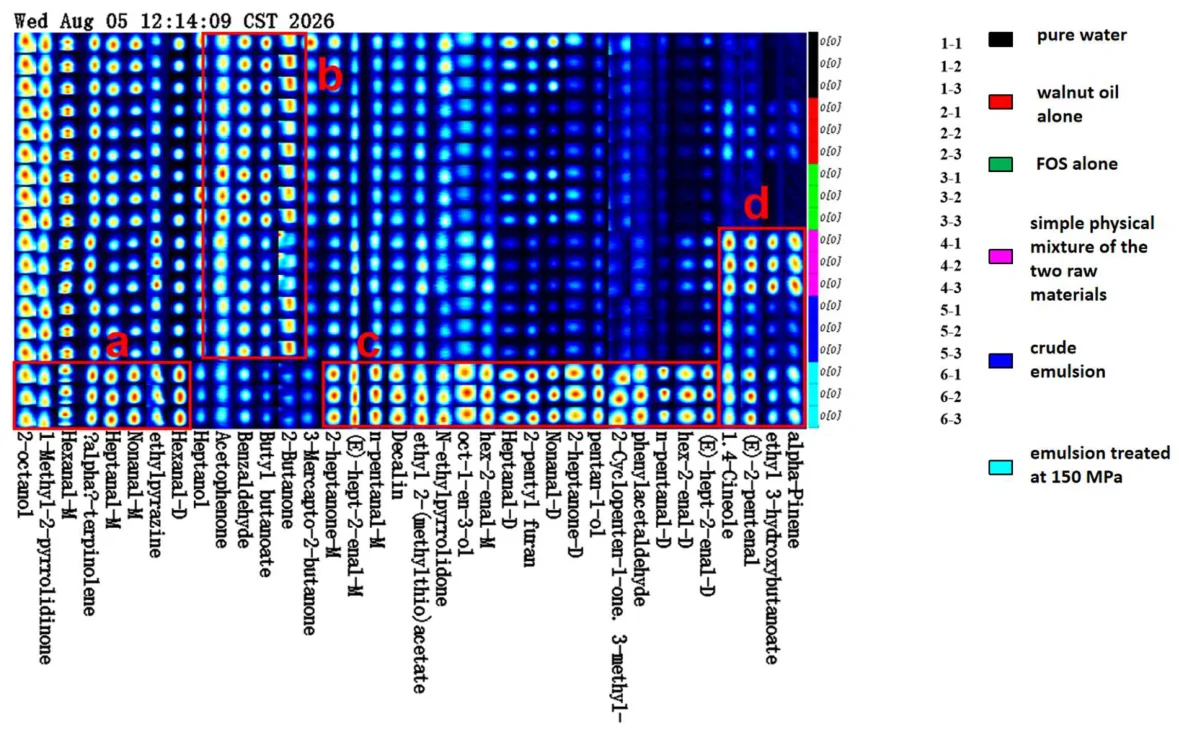
Gallery Plot fingerprints of VOCs in cooked rice supplemented with different additives detected by GC–IMS.

GC-IMS was applied to detect VOCs and evaluate the authenticity and quality of food matrices. This technique integrates the superior separation performance of gas chromatography and the ultrahigh sensitivity of ion mobility spectrometry, enabling efficient separation and identification of trace-level VOCs in samples [36,37]. In the present study, GC–IMS was used to characterize differences in VOC profiles of cooked rice supplemented with various additives. To visually distinguish the variations among treatments, the VOC fingerprints of all samples were further compared via the Gallery Plot plugin, as illustrated in Figure 19, and a total of 34 characteristic signal peaks were identified. Each horizontal row corresponds to all selected volatile peaks within one sample, while each vertical column represents the identical VOC across different treatments. Color intensity reflects the relative concentration of VOCs: red indicates a higher relative content, whereas blue denotes a lower concentration [38].

Region a (enrichment zone for aldehydes, pyrazines, and alcohols; representative compounds: 2-octanol, hexanal, heptanal, nonanal, ethylpyrazine, etc.). The pure water control (Group 1) exhibited the weakest overall signal intensity, with extensive blue areas across this region, indicating the lowest levels of aldehydes, pyrazines, and esters, which only provided the faint basic flavor of plain cooked rice. In Group 2 (walnut oil alone), the signals of hexanal, heptanal, and nonanal were markedly elevated, originating from oxidative degradation of unsaturated fatty acids in walnut oil; the resulting aliphatic aldehydes imparted grassy and nutty aromatic notes to the rice. Group 3 (FOS alone) showed a slight increase in signal intensity, as mild Maillard reactions occurred during thermal processing of fructooligosaccharides, generating small amounts of aldehydes and pyrazines. In Group 4 (simple physical mixture of the two raw materials), the abundances of aldehydes and pyrazines increased compared with Groups 1, 2, and 3; however, the limited integration between oil and aqueous phases restricted the synergistic interplay between lipid oxidation and Maillard reaction, resulting in substantially lower accumulation levels than those observed in the crude emulsion group. Group 5 (crude emulsion) exhibited further enhanced abundances of aldehydes and pyrazines, benefiting from the dual synergistic aroma-generating pathways of walnut oil lipid oxidation and FOS-mediated Maillard reaction. Group 6 (emulsion treated at 150 MPa) achieved the strongest warm-colored signals in Region a: high-pressure microfluidization drastically refined emulsion droplets and greatly expanded the oil–water interfacial area, simultaneously accelerating lipid oxidation and FOS-mediated Maillard reactions, thereby producing significantly higher contents of nutty and roasted pyrazine flavors than all other groups.

Region b (enrichment zone for ketones and aldehydes; representative compounds: 2-butanone, butyl butanoate, benzaldehyde, acetophenone, and heptanol). Group 1 predominantly exhibited a blue background across this region, with the five target flavor compounds showing weak spot brightness and low abundance. Only trace background flavors generated during the rice cooking process were detectable, contributing minimally to the sensory perception of fruity, nutty, and alcoholic notes. In Group 2, the signals of heptanol and benzaldehyde showed slight increases. Heptanol could impart fatty, grassy, and alcoholic notes, while benzaldehyde provided almond-like nutty aromas. However, ketones and esters did not exhibit significant enhancement, resulting in a relatively narrow flavor profile. Group 3 only displayed modest increases in benzaldehyde and acetophenone, primarily derived from mild Maillard reactions during thermal processing of fructooligosaccharides, whereas butyl butanoate and 2-butanone showed almost no noticeable enrichment in the system. In Group 4, only slight accumulation of the five compounds was observed. Due to the simple physical mixing of walnut oil and FOS without sufficient oil–water contact, the synergistic interplay between lipid oxidation and Maillard reactions was suppressed. Although heptanol and benzaldehyde signals were slightly higher than those in Groups 1, 2, and 3, the insufficient enrichment of butyl butanoate, acetophenone, and 2-butanone made it difficult to generate adequate complex fruity and roasted aromas. In Group 5, the spot brightness of all five compounds increased simultaneously. The emulsion structure improved the contact conditions between lipids and carbohydrates, promoting synergistic lipid oxidation and Maillard reactions, thereby achieving better accumulation of alcoholic, nutty, and fruity notes than Group 4.

Region c (enrichment zone for unsaturated aliphatic alkenals, saturated aliphatic aldehydes, aromatic aldehydes, furan derivatives, lactams, and alicyclic alkanes; representative compounds: N-ethylpyrrolidone, 2-pentylfuran, decalin, 3-methyl-2-cyclopenten-1-one (caramel roasted aroma), phenylacetaldehyde (floral and honey-like scent), n-pentanal-D, etc.). In Group 6, the oil–water contact area was significantly enlarged, enabling simultaneous enrichment of multiple high-quality flavor compounds. Specifically, 1-octen-3-ol contributed fresh mushroom-like notes; cyclopentenones imparted caramel roasted aromas; phenylacetaldehyde provided honey-sweet floral notes; 1-pentanol delivered alcoholic and light fruity notes; 2-heptanone (dimer) released fruity and subtle creamy aromas; and nonanal (dimer) contributed fatty and citrus-like fresh notes. These compounds collectively constructed a multi-layered aromatic profile for the cooked rice, making this region a key differentiator between Group 6 and the other groups.

Region d (enrichment zone for terpene characteristic components; representative compounds: α-pinene and other terpenes). As shown in the fingerprint, high-brightness spots in Region d were predominantly concentrated in Group 4, whereas Groups 1, 2, 3, and 5 exhibited weak signals with a predominantly blue background in this region. Group 6 showed lower signal intensity than Group 4. In Group 4 (simple physical mixture of walnut oil and FOS), the two raw materials were unevenly distributed and poorly integrated within the rice matrix, allowing most terpenes to accumulate in their free state, thus resulting in prominent spot brightness. In contrast, in Group 6 (prepared via high-pressure microfluidization), the signal of free terpenes decreased, as terpene components were uniformly dispersed and embedded into the interior of the rice starch matrix with a more homogeneous spatial distribution. This enabled slow and sustained aroma release during cooking and consumption, effectively prolonging the aroma persistence of the cooked rice.

In summary, the single addition of either walnut oil or FOS only generated a single category of volatile aromas, resulting in limited flavor improvement. The simple physical mixture of walnut oil and FOS only enriched free terpenes without sufficient synergistic generation of complex aromas. The crude emulsion without homogenization exhibited only a moderate flavor enhancement due to insufficient oil–water interfacial contact. In contrast, the oil-in-water walnut oil–FOS emulsion treated with 150 MPa high-pressure microfluidization effectively enlarged the oil–water interfacial area, significantly enriching nutty lipid-derived aromas, caramel roasted notes, milky and honey-like volatile flavor compounds, while maintaining excellent flavor stability. This approach maximized the overall intensity and layered complexity of the cooked rice aroma.

## 4. Discussion

The present study has successfully demonstrated the feasibility of fabricating walnut oil–FOS nano-emulsions via high-pressure microfluidization and confirmed their synergistic efficacy in improving the texture, color, and flavor profiles of cooked rice. Nevertheless, several aspects remain to be further investigated. First, the pressure-dependent reduction in antioxidant activity observed at ≥120 MPa warrants attention regarding its potential influence on the long-term oxidative stability of the final product. Second, the current findings are based on a single rice variety, and the applicability of the optimized emulsion to other cultivars or processing conditions requires further confirmation.

To address these limitations, future research could focus on enhancing the oxidative stability of the emulsion system through appropriate technological approaches. For example, the addition of 0.02–0.05% natural antioxidants, such as rosemary extract, vitamin E, or tea polyphenols, to the emulsion system could be explored as a feasible strategy to offset the loss of antioxidant capacity caused by high-pressure microfluidization. Systematic storage studies under various conditions would also help to elucidate the actual stability and quality retention of the emulsion and the cooked rice over extended periods. Furthermore, validation using different rice varieties and scale-up trials would be valuable to establish the broader applicability and industrial potential of the developed system.

## 5. Conclusions

In this study, sucrose fatty acid ester (SE) was identified as the optimal emulsifier for walnut oil–fructooligosaccharide emulsions. The optimal formulation was a water-to-oil ratio of 6:4, 4% SE, and 150 MPa microfluidization, yielding the smallest droplet size (255.07 nm), highest emulsion stability index (~340 min), and most uniform droplet distribution. When applied to rice cooking, the 150 MPa nano-emulsion delivered the lowest water loss (51%), highest lightness (L* = 79.62), lowest hardness (3297.22 g), and highest springiness (0.74), alongside the most abundant volatile organic compounds (e.g., aldehydes, pyrazines, ketones, and esters). In contrast, single additives, physical mixtures, and crude emulsions showed textural or flavor defects, highlighting the necessity of emulsification and homogenization. Mechanistically, the synergistic improvement is attributed to starch–lipid complexation and a uniform polysaccharide–lipid network that inhibits retrogradation.

However, the pressure-induced antioxidant loss at ≥120 MPa and its effect on long-term oxidative stability were not assessed, nor was the applicability to other rice varieties. Despite these limitations, this study provides a practical strategy and theoretical basis for using walnut oil–FOS nano-emulsions as a clean-label approach to improve cooked rice quality and support staple food functionalization.

## Figures and Tables

**Table 1:** Color attributes (L*, a*, and b*) of cooked rice supplemented with different additives.

Medium/Parameters	L*	a*	b*
Water (control)	68.0267 ± 0.18 d	−1.8667 ± 0.01 d	6.3033 ± 0.06 a
Walnut oil	73.8967 ± 0.09 b	−2.1100 ± 0.01 e	2.6767 ± 0.03 e
FOS	68.0767 ± 0.51 d	−1.6267 ± 0.01 a	4.9233 ± 0.05 c
Walnut oil and FOS	69.9267 ± 0.77 c	−1.7600 ± 0.00 c	5.31 ± 0.02 b
Crude emulsion	70.3700 ± 0.87 c	−1.8567 ± 0.02 d	3.4300 ± 0.15 d
Microfluidized emulsion (150 MPa)	79.6200 ± 1.29 a	−1.7267 ± 0.02 b	4.8100 ± 0.12 c

Different letters within the same column denote significant differences (*p* < 0.05), while the same letter indicates no significant difference.

**Table 2:** Textural profile analysis (TPA) parameters of cooked rice supplemented with different additives.

Medium/Parameters	Hardness (g)	Adhesiveness (g·s)	Springiness	Chewiness (g)
Water (control)	6502.44 ± 177.57 a	1562.51 ± 125.13 a	0.20 ± 0.01 b	514.40 ± 15.41 ab
Walnut oil	4022.51 ± 158.14 b	761.94 ± 15.79 c	0.27 ± 0.00 b	509.57 ± 35.47 ab
FOS	3595.43 ± 152.28 cd	1599.90 ± 100.96 a	0.66 ± 0.05 a	523.08 ± 34.42 a
Walnut oil and FOS combined group	3635.72 ± 245.42 c	1465.60 ± 85.97 ab	0.71 ± 0.14	495.16 ± 51.32 bc
Crude emulsion	3387.27 ± 204.11 cd	1614.10 ± 399.87 a	0.67 ± 0.02 a	435.37 ± 17.96 bc
Microfluidized emulsion (150 MPa)	3297.22 ± 44.89 cd	1172.03 ± 14.96 b	0.74 ± 0.08 a	453.01 ± 30.53 bc

Different letters within the same column denote significant differences (*p* < 0.05), while the same letter indicates no significant difference.

## Data Availability

The original contributions presented in this study are included in the article. Further inquiries can be directed to the corresponding author.
